# Lefamulin efficacy and safety in a pooled phase 3 clinical trial population with community-acquired bacterial pneumonia and common clinical comorbidities

**DOI:** 10.1186/s12890-021-01472-z

**Published:** 2021-05-08

**Authors:** Thomas M. File, Elizabeth Alexander, Lisa Goldberg, Anita F. Das, Christian Sandrock, Susanne Paukner, Gregory J. Moran

**Affiliations:** 1 Summa Health, Akron, OH USA; 2 Nabriva Therapeutics US, Inc., Fort Washington, PA USA; 3 Das Consulting, Guerneville, CA USA; 4 UC Davis School of Medicine, Sacramento, CA USA; 5 Nabriva Therapeutics GmbH, Vienna, Austria; 6 Olive View-UCLA Medical Center, Los Angeles, CA USA; 7 Present Address: Vir Biotechnology, Inc., 499 Illinois Street, Suite 500, San Francisco, CA 94158 USA

**Keywords:** Antibiotic, Clinical response, Lefamulin, Pleuromutilin, Pneumonia

## Abstract

**Background:**

Lefamulin, a first-in-class pleuromutilin antibiotic approved for intravenous and oral use in adults with community-acquired bacterial pneumonia (CABP), was noninferior to moxifloxacin in the Lefamulin Evaluation Against Pneumonia (LEAP) 1 intravenous-to-oral switch study and the LEAP 2 oral-only study. Using pooled LEAP 1/2 data, we examined lefamulin efficacy/safety overall and within subgroups of patients presenting with comorbidities typical in CABP management.

**Methods:**

In LEAP 1, adults with CABP were randomized to receive intravenous lefamulin (150 mg every 12 h) for 5‒7 days or moxifloxacin (400 mg every 24 h) for 7 days, with optional intravenous-to-oral switch if predefined improvement criteria were met. In LEAP 2, adults with CABP were randomized to receive oral lefamulin (600 mg every 12 h) for 5 days or moxifloxacin (400 mg every 24 h) for 7 days. Both studies assessed early clinical response (ECR) at 96 ± 24 h after first study drug dose and investigator assessment of clinical response (IACR) at test-of-cure (5‒10 days after last dose). Pooled analyses of the overall population used a 10% noninferiority margin.

**Results:**

Lefamulin (n = 646) was noninferior to moxifloxacin (n = 643) for ECR (89.3% vs 90.5%, respectively; difference  − 1.1%; 95% CI  − 4.4 to 2.2); IACR success rates at test-of-cure were similarly high (≥ 85.0%). High efficacy with both lefamulin and moxifloxacin was also demonstrated across all well-represented patient subgroups, including those with advanced age, diabetes mellitus, a history of cardiovascular diseases (e.g., hypertension, congestive heart failure, or arrhythmia) or chronic lung diseases (e.g., asthma or chronic obstructive pulmonary disease), elevated liver enzymes, or mild-to-moderate renal dysfunction. No new safety signals were identified.

**Conclusions:**

Lefamulin may provide a valuable intravenous/oral monotherapy alternative to fluoroquinolones or macrolides for empiric treatment of patients with CABP, including cases of patients at risk for poor outcomes due to age or various comorbidities.

***Trial registration*:**

ClinicalTrials.gov LEAP 1 (NCT02559310; Registration Date: 24/09/2015) and LEAP 2 (NCT02813694; Registration Date: 27/06/2016).

**Supplementary Information:**

The online version contains supplementary material available at 10.1186/s12890-021-01472-z.

## Background

Pneumonia, a leading cause of US hospitalizations and infection-related deaths [1–3], is associated with substantial morbidity and mortality [4]. Pneumonia-associated economic burden is considerable, with annual costs estimated at €10.1 billion in Europe [5] and exceeding $17 billion in the United States [6]. Pneumonia prognoses can range from rapid resolution to death [7], and clinical management of community-acquired bacterial pneumonia (CABP) can be complicated by many factors [1]. CABP incidence and impact are greater in older versus younger individuals, and older patients with CABP often present with challenges (e.g., increased resistance rates, polypharmacy/drug interactions) that can impede treatment efficacy and safety [8, 9]. Comorbidities such as chronic obstructive pulmonary disease (COPD), congestive heart failure (CHF), or diabetes mellitus (DM) increase the risk of severe CABP and may aggravate clinical symptoms and complicate management [9, 10]. Underlying cardiac or liver disease also increase the risk of potential cardiac or liver toxicities, respectively, associated with CABP antimicrobials [11–13].

Increasingly, new CABP treatment options are needed owing to rising antibacterial resistance rates and undesirable risks/adverse effects associated with current treatments, including allergic reactions and fluoroquinolone-associated disability (e.g., tendon injury, aortic rupture, glucose homeostasis imbalance, and neurocognitive effects, especially in older patients) [1, 11, 14–16]. Lefamulin, a first-in-class pleuromutilin antibiotic that selectively inhibits bacterial protein synthesis [17, 18], is approved for intravenous (IV) and oral use in adults with CABP [19]. In adults with CABP, lefamulin demonstrated noninferiority to moxifloxacin in the IV-to-oral switch Lefamulin Evaluation Against Pneumonia (LEAP) 1 study [20] and the oral-only LEAP 2 study [21]. Lefamulin has demonstrated potent in vitro activity against a global collection of typical (eg, *Streptococcus pneumoniae, Staphylococcus aureus, Haemophilus influenzae*, *Moraxella catarrhalis)* and atypical (e.g., *Mycoplasma pneumoniae, Legionella pneumophila, Chlamydophila pneumoniae*) CABP-causative pathogens, including antimicrobial-resistant strains [22, 23]; lefamulin has also demonstrated clinical efficacy against these and other CABP pathogens [20, 21]. Moreover, lefamulin has demonstrated weak activity against *Bacteroides fragilis* group strains and *Enterobacteriaceae*, suggesting decreased potential for disruption to gastrointestinal (GI) microbiota by IV lefamulin (Data on file, Nabriva Therapeutics, Vienna, Austria). In contrast to other common CABP therapies, the suitability of lefamulin for oral administration and its targeted antimicrobial activity [19] may position it as an attractive option for use in patients with advanced age, various comorbid conditions, increased disease severity, or drug allergies. Therefore, data from the 2 pivotal phase 3 LEAP studies in adults with CABP were pooled to further examine lefamulin efficacy and safety overall and within various patient subgroups.

## Methods

### Study design and participants

The LEAP 1 (NCT02559310; Registration Date: 24/09/2015) [20] and LEAP 2 (NCT02813694; Registration Date: 27/06/2016) [21] studies were double-blind, double-dummy, parallel-group trials evaluating lefamulin versus moxifloxacin in adults with moderate to severe CABP. Before study initiation, participating centers obtained study approval from appropriate ethics committees/institutional review boards (Additional file 1: Appendices 1‒2), and patients provided written informed consent. Trials were compliant with the ethical principles of the Declaration of Helsinki, Good Clinical Practice guidelines, and local laws/regulations.

Detailed methods for both studies have been published elsewhere [20, 21]. Briefly, randomization (1:1) was stratified in both studies by Pneumonia Outcomes Research Team (PORT) risk class (III vs IV/V [LEAP 1]; II vs III/IV [LEAP 2]), geographic region (US vs ex-US), and receipt of a single dose of a short-acting antibacterial for CABP before randomization (yes vs no). In LEAP 1, patients received IV lefamulin 150 mg every 12 h (q12h) for 5‒7 days or moxifloxacin 400 mg every 24 h (q24h) for 7 days. If methicillin-resistant *S. aureus* (MRSA) was suspected at screening, blinded IV linezolid (600 mg q12h) was added to moxifloxacin, or linezolid placebo was added to lefamulin.

In LEAP 1, patients could switch to oral therapy (lefamulin 600 mg q12h or moxifloxacin 400 mg q24h) after 6 IV doses (~ 3 days) if predefined improvement criteria were met. The original study protocol indicated a 5-day lefamulin treatment period (10 days in patients with CABP due to *L. pneumophila* or MRSA or in patients with *S. pneumoniae* and bacteremia); however, this was later adjusted to 7 days (except in confirmed MRSA cases, which continued to receive 10 days of treatment) to reduce medication errors and limit study site burden. In LEAP 2, patients received oral lefamulin 600 mg q12h (5 days) or moxifloxacin 400 mg q24h (7 days).

Patients eligible for enrollment included adults with radiographically diagnosed pneumonia, PORT risk class III‒V (LEAP 1) or II‒IV (LEAP 2), acute onset of  ≥ 3 CABP symptoms (e.g., dyspnea),  ≥ 2 vital sign abnormalities (e.g., tachycardia), and ≥ 1 other clinical sign or laboratory finding of CABP (e.g., hypoxemia). Exclusion criteria included receipt of  > 1 dose of a short-acting CABP oral/IV antibacterial within 72 h before randomization, being at risk for major cardiac events or dysfunction, significant hepatic disease, and severely impaired renal function. Of note, not all patients with comorbidities were excluded; patients with DM, asthma/COPD, non-major cardiac events, elevated liver enzymes, and mild-to-moderate renal dysfunction were included and well represented in the LEAP 1 and LEAP 2 trials.

### Study outcomes

In both studies, the US Food and Drug Administration (FDA) primary efficacy endpoint was early clinical response (ECR) at 96 ± 24 h after first study drug dose in the intent-to-treat (ITT) population (all randomized patients). Patients were programmatically classified as ECR responders if they were alive, showed improvement in ≥ 2 CABP symptoms, had no CABP symptom worsening, and received no nonstudy antibiotic for CABP treatment. Achievement of ECR criteria was also assessed at each study day, end of treatment (EOT; within 2 days after last study drug dose), test of cure (TOC; 5**‒**10 days after last study drug dose), and late follow-up (LFU; days 27**‒**34).

Secondary endpoints (European Medicines Agency primary endpoints) were investigator assessment of clinical response (IACR) at TOC in the modified ITT (mITT) and clinically evaluable (CE) populations. The mITT population included all randomized patients who received any study drug. The CE population comprised patients with no indeterminate clinical response, ≥ 48 h of study drug (unless patient died before 48 h), receipt of no nonstudy antibacterial potentially effective against CABP pathogens (unless administered for clinical failure), and no additional efficacy confounding factors. Investigator assessment classified patient response as a success if CABP signs/symptoms were resolved or improved such that no additional antibiotic was administered for the current CABP episode or as a failure if nonstudy antibiotics were administered or patient died from any cause. IACR success rates were also assessed at EOT and LFU.

Additional analyses included ECR and IACR assessments in subgroups based on baseline demographics and disease characteristics (Additional file 2: Table 1). Safety was assessed in all randomized patients who received any amount of study drug (safety population). Treatment-emergent adverse events (TEAEs) were defined as adverse events (AEs) that started/worsened during or after first study drug administration and were monitored at all study visits and by patient reporting. Additional safety assessments included vital signs, laboratory tests, and triplicate 12-lead electrocardiograms, which were performed within a 5-min interval at screening and on days 1 and 3 (LEAP 1) or days 1 and 4 (LEAP 2) to capture changes at the estimated maximum observed plasma concentration of study drug.

### Statistical analyses

Pooled ECR and IACR analyses were evaluated using a 10% noninferiority margin; lefamulin noninferiority versus moxifloxacin was concluded if the lower 95% CI limit for the treatment difference exceeded  − 10%. Treatment differences were weighted by study. The 95% CI was calculated using the Miettinen-Nurminen method [24], adjusted for study (ECR and IACR) and receipt of a prior single-dose short-acting antibiotic (IACR only), with the inverse variance of the effect size used for stratum weights. For subgroup analyses, descriptive CIs were determined but no noninferiority conclusions were made. If the treatment difference and CI were not estimable using these methods, observed treatment differences were reported and 95% CIs were calculated via unadjusted continuity-corrected Z-test.

## Results

### Patients

The pooled ITT population included 1289 patients randomized to lefamulin (n = 646) or moxifloxacin (n = 643). Detailed patient dispositions have been published elsewhere [20, 21]. Baseline demographic and disease characteristics were generally well balanced between treatment groups and reflected the general patient population with CABP (Table 1). Overall, 40.1% of patients were aged ≥ 65 years, 55.6% were male, 51.4% had impaired renal function, and 70.8% had PORT risk class ≥ III. Common comorbidities included hypertension (38.9%), asthma/COPD (18.0%), DM (13.0%), and CHF (10.2%). As expected, patients with PORT risk class IV/V were older and more likely to have comorbidities than patients with PORT risk class III. Among PORT risk class III and IV/V patients, 16.3% and 42.0% were aged ≥ 75 years, respectively. Compared with PORT risk class III patients, more PORT risk class IV/V patients had comorbidities such as renal impairment (53.5% vs 73.9%, respectively), hypertension (41.5%, 52.1%), asthma/COPD (16.4%, 29.4%), and DM (12.1%, 22.7%).

**Table 1:** Demographic and baseline characteristics (pooled ITT population)

Parameter	Lefamulin (n = 646)	Moxifloxacin (n = 643)	Overall (N = 1289)
Age, y, mean (SD)	58.9 (16.5)	58.5 (15.7)	58.7 (16.1)
Age group, years, n (%)			
18**‒**64	378 (58.5)	394 (61.3)	772 (59.9)
65**‒**74	152 (23.5)	145 (22.6)	297 (23.0)
75–84	90 (13.9)	87 (13.5)	177 (13.7)
≥ 85	26 (4.0)	17 (2.6)	43 (3.3)
Male, n (%)	377 (58.4)	340 (52.9)	717 (55.6)
White, n (%)	513 (79.4)	509 (79.2)	1022 (79.3)
BMI, kg/m^2^, mean (SD)	26.5 (5.8)	26.4 (6.0)	26.5 (5.9)
PORT risk class,* n (%)			
I/II	184 (28.5)	192 (29.9)	376 (29.2)
III	341 (52.8)	334 (51.9)	675 (52.4)
IV/V	121 (18.7)	117 (18.2)	238 (18.5)
CURB-65 score,^†^ n (%)			
0**‒**2	610 (94.4)	604 (93.9)	1214 (94.2)
3**‒**5	36 (5.6)	39 (6.1)	75 (5.8)
Minor ATS severity criteria,^‡^ n (%)	85 (13.2)	85 (13.2)	170 (13.2)
Modified ATS severity criteria,^§^ n (%)	53 (8.2)	57 (8.9)	110 (8.5)
Prior antibiotic use,^||^ n (%)	147 (22.8)	145 (22.6)	292 (22.7)
Baseline renal status,^¶^ n (%)			
Normal function	311 (48.1)	312 (48.5)	623 (48.3)
Mild impairment	201 (31.1)	192 (29.9)	393 (30.5)
Moderate impairment	125 (19.3)	132 (20.5)	257 (19.9)
Severe impairment	7 (1.1)	6 (0.9)	13 (1.0)
Missing	2 (0.3)	1 (0.2)	3 (0.2)
Medical history,^#^ n (%)			
Smoking history	284 (44.0)	242 (37.6)	526 (40.8)
Hypertension	248 (38.4)	253 (39.3)	501 (38.9)
Baseline liver enzyme elevation	119 (18.4)	144 (22.4)	263 (20.4)
Asthma/COPD	119 (18.4)	113 (17.6)	232 (18.0)
Diabetes mellitus	80 (12.4)	88 (13.7)	168 (13.0)
Congestive heart failure	57 (8.8)	75 (11.7)	132 (10.2)
Arrhythmia	43 (6.7)	30 (4.7)	73 (5.7)
SIRS,^**^ n (%)	621 (96.1)	609 (94.7)	1230 (95.4)
Bacteremia, n (%)	13 (2.0)	12 (1.9)	25 (1.9)

*ATS* American Thoracic Society, *BMI* body mass index, *BUN* blood urea nitrogen, *COPD* chronic obstructive pulmonary disease, *CrCl* creatinine clearance, *ITT* intent to treat, *PORT* Pneumonia Outcomes Research Team, *SIRS* systemic inflammatory response syndrome, *WBC* white blood cell (count)

*PORT risk class calculated programmatically using site data reported in the electronic case report form was not always consistent with the site-reported PORT risk class used for enrollment/stratification; consequently, 3 patients with PORT risk class I (lefamulin, n = 1; moxifloxacin, n = 2) were enrolled

^†^Defined as confusion of new onset, BUN > 19 mg/dL, respiratory rate ≥ 30 breaths/min, systolic blood pressure < 90 mm Hg or diastolic blood pressure ≤ 60 mm Hg, and age ≥ 65 years

^‡^Defined as baseline presence of ≥ 3 of the following 9 criteria: respiratory rate ≥ 30 breaths/min, O_2_ saturation < 90% or PaO_2_ < 60 mm Hg, BUN ≥ 20 mg/dL, WBC < 4000 cells/mm^3^, confusion, multilobar infiltrates, platelets < 100,000 cells/mm^3^, temperature < 36 °C, or systolic blood pressure < 90 mm Hg [40]

^§^Defined as baseline presence of ≥ 3 of the following 6 criteria: respiratory rate ≥ 30 breaths/min, SpO_2_/FiO_2_ < 274 where SpO_2_/FiO_2_ = 64 + 0.84 (PaO_2_/FiO_2_), BUN ≥ 20 mg/dL, confusion, age ≥ 65 years, or multilobar infiltrates [41]

^||^Patients received a single dose of short-acting systemic antibacterial medication ≤ 72 h before randomization; randomization was stratified and capped such that ≤ 25% of the total ITT population met these criteria

^¶^Defined as normal (CrCl ≥ 90 mL/min), mild (CrCl 60 to < 90 mL/min), moderate (CrCl 30 to < 60 mL/min), and severe (CrCl < 30 mL/min)

^#^See Additional file 2: Supplemental Table 1 for definitions of medical history terms

**Defined as baseline presence of ≥ 2 of the following 4 criteria: temperature < 36 °C or > 38 °C; heart rate > 90 bpm; respiratory rate > 20 breaths/min; and WBC < 4000 cells/mm^3^, WBC > 12,000 cells/mm^3^, or immature polymorphonuclear neutrophils > 10%

### Clinical response/success

**Fig. 1:**
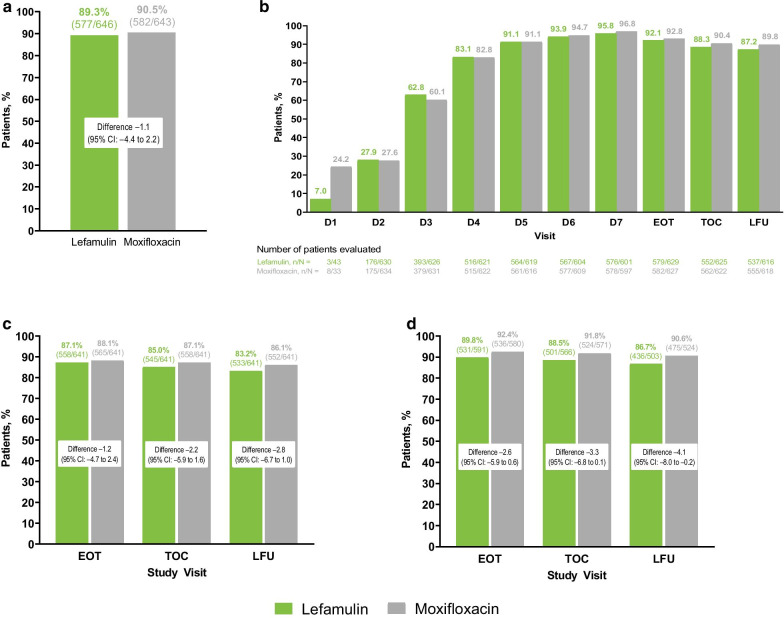
**a** ECR response in the pooled ITT population, **b** patients meeting ECR criteria by visit in the pooled ITT population, and IACR success by visit in the pooled **c** mITT and **d** CE populations. *CE* clinically evaluable, *ECR* early clinical response, *EOT* end of treatment, *IACR* investigator assessment of clinical response, *ITT* intent to treat, *LFU* late follow-up, *mITT* modified ITT, *TOC *test of cure

**Fig. 2:**
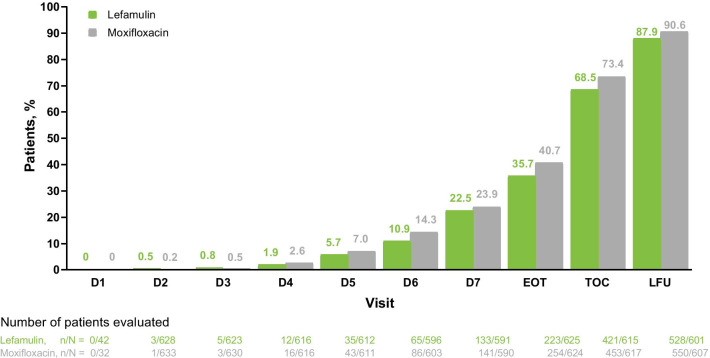
Patients with resolution of all baseline clinical signs and symptoms* of CABP by visit (pooled ITT population). *Dyspnea, cough, purulent sputum production, and chest pain. *CABP* community-acquired bacterial pneumonia, *EOT* end of treatment, *ITT* intent to treat, *LFU* late follow-up, *TOC* test of cure

In the pooled ITT population, ECR rates were high (lefamulin 89.3%; moxifloxacin 90.5%; difference − 1.1; 95% CI − 4.4 to 2.2), and lefamulin was noninferior to moxifloxacin (Fig. 1a). Most patients (> 60%) met ECR criteria by day 3, with > 80% of patients in both treatment groups meeting ECR criteria by day 4; further increases through day 7 and sustained efficacy through LFU were also observed (Fig. 1b). Similarly, proportions of patients with resolution of all baseline CABP signs and symptoms also increased over time in both treatment groups (Fig. 2).

Lefamulin was also noninferior to moxifloxacin for IACR success (mITT and CE populations), with high and similar rates at TOC (Fig. 1c, d) for both lefamulin and moxifloxacin. IACR success rates at EOT and LFU were similarly high (> 83%) and consistent between treatment groups.

**Fig. 3:**
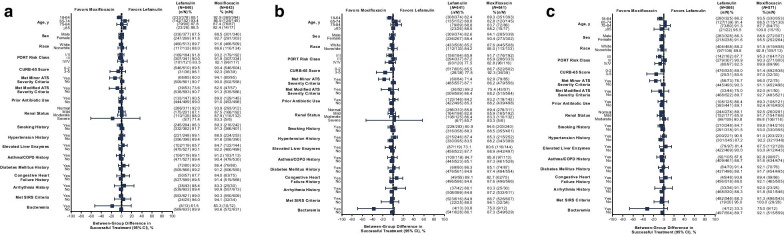
Subgroup analysis of **a** ECR in the pooled ITT population and IACR at TOC in the pooled **b** mITT and **c** CE populations. See Table 1 footnotes for subgroup definitions. *ATS* American Thoracic Society, *CE* clinically evaluable, *COPD* chronic obstructive pulmonary disease, *ECR* early clinical response, *IACR* investigator assessment of clinical response, *ITT* intent to treat, *mITT* modified ITT, *PORT* Pneumonia Outcomes Research Team, *SIRS* systemic inflammatory response syndrome

Lefamulin and moxifloxacin demonstrated high and similar ECR rates across various baseline demographic and disease characteristics, including patients with advanced age or comorbidities (asthma/COPD, DM, hypertension, CHF, arrhythmia, elevated liver enzymes) and regardless of PORT risk class (Fig. 3a). IACR success rates at TOC for both treatment groups were similarly high across most subgroups (Fig. 3b, c).

### Safety

Within the pooled safety population (n = 1282), mean (SD) study drug exposure was 4.9 (1.5) days for lefamulin (n = 641) and 6.8 (1.1) days for moxifloxacin (n = 641) overall. This included patients who received IV treatment only (LEAP 1 patients; mean duration of 5.9 [2.0] days with lefamulin [n = 273] and 5.4 [1.9] days for moxifloxacin [n = 273]) or oral treatment only (LEAP 2 patients; 4.7 [1.1] days with lefamulin [n = 472] and 5.9 (1.8) days with moxifloxacin [n = 489]).

Overall TEAE rates were similar with lefamulin (34.9%) and moxifloxacin (30.4%; Table 2); most TEAEs were mild to moderate in severity and few patients (< 5%) experienced severe TEAEs. Treatment-related TEAEs were reported in 15.4% (lefamulin) and 10.6% (moxifloxacin) of patients, serious TEAEs in 5.6% and 4.8%, and TEAEs leading to study drug discontinuation in 3.1% and 3.3%. Over the entire study duration, 19 patients experienced TEAEs leading to death (lefamulin n = 11, moxifloxacin n = 8; Table 2). Among patients with common comorbidities, lefamulin and moxifloxacin demonstrated overall similar TEAE profiles to the overall population (Additional file 2: Tables 1‒3). Regardless of treatment group, the percentage of patients with severe TEAEs, serious TEAEs, and TEAEs leading to death increased with advancing age or PORT risk class.

**Table 2:** Overall summary of TEAEs (pooled safety population)

Patients, n (%)	Lefamulin (n = 641)	Moxifloxacin (n = 641)
All TEAEs*	224 (34.9)	195 (30.4)
Mild	119 (18.6)	117 (18.3)
Moderate	78 (12.2)	55 (8.6)
Severe	27 (4.2)	23 (3.6)
Related TEAEs^†^	99 (15.4)	68 (10.6)
Serious TEAEs	36 (5.6)	31 (4.8)
TEAEs leading to study drug discontinuation^‡^	20 (3.1)	21 (3.3)
TEAEs leading to death by study day 28^§^	8 (1.2)	7 (1.1)
TEAEs leading to death (over entire study duration)^||^	11 (1.7)	8 (1.2)
TEAEs by preferred term in ≥ 2% of patients		
Diarrhea	47 (7.3)	25 (3.9)
Nausea	27 (4.2)	13 (2.0)
Vomiting	15 (2.3)	4 (0.6)

*AE* adverse event, *COPD* chronic obstructive pulmonary disease, *MedDRA* Medical Dictionary for Regulatory Activities, *PORT* Pneumonia Outcomes Research Team, *TEAE* treatment-emergent AE

*AEs with unknown start date, or partial date such that it could not be determined if they started on or after first study drug administration, were categorized as TEAEs; AEs were classified using the MedDRA version 20.0

^†^Related TEAEs were defined as TEAEs that were considered “definitely,” “probably,” or “possibly” related to study drug by the investigator. If the relationship for a TEAE was missing, it was considered “related.” Patients with multiple events in each category were counted only once in that category

^‡^A patient could have > 1 TEAE leading to study drug discontinuation

^§^Assessed in the intent-to-treat population (lefamulin, n = 646; moxifloxacin, n = 643)

^||^Three patients in the lefamulin group had a TEAE leading to death after study day 28: 1 patient (aged 87 years; PORT risk class III; liver enzyme elevation and moderate renal impairment [creatinine clearance 30 to < 60 mL/min] at baseline; history of hypertension and COPD) died on study day 32 from sepsis (first reported on study day 31); 1 patient (aged 80 years; PORT risk class III; baseline moderate renal impairment; history of hypertension and COPD) died on study day 57 from endocarditis (first reported on study day 24); and 1 patient (aged 70 years; PORT risk class II; baseline moderate renal impairment; history of hypertension and COPD) died on study day 271 from acute myeloid leukemia (first reported on study day 269). One patient in the moxifloxacin group (aged 26 years; PORT risk class IV) had a TEAE leading to death on study day 48 due to testicular seminoma (first reported on study day 21)

The most frequently reported TEAEs in the lefamulin (13.1% [84/641]) and moxifloxacin (10.1% [65/641]) groups were GI. The most common individual TEAEs were diarrhea (7.3% [lefamulin] and 3.9% [moxifloxacin]), nausea (4.2%, 2.0%), and vomiting (2.3%, 0.6%) (Table 2), and most were mild to moderate in severity. Differences between the 2 treatment groups were driven primarily by GI events associated with oral dosing in the LEAP 2 study. Few patients discontinued study drug because of GI TEAEs (lefamulin, 0.5% [3/641]; moxifloxacin, 0.2% [1/641]). One case of *Clostridium difficile* infection was reported in a LEAP 2 patient successfully treated with lefamulin; onset occurred ~ 1 week after completing 5 days of lefamulin treatment, patient remained hospitalized, and infection resolved after oral vancomycin treatment.

Treatment-emergent adverse events (TEAEs) in the Medical Dictionary for Regulatory Activities system organ class (SOC) of cardiac disorders occurred in 2.5% (lefamulin) and 3.1% (moxifloxacin) of patients (Additional file 2: Table 4). After dosing, mean QT intervals corrected according to Fridericia (QTcF) increased from baseline in both treatment groups, although the mean (SD) maximum change from baseline was numerically smaller with lefamulin (16.9 [16.9] ms) than moxifloxacin (19.3 [17.7] ms). No associated cardiac arrhythmias of concern were observed. Among patients with history of hypertension or arrhythmia and those aged ≥ 65 years, TEAEs in the cardiac disorders SOC were reported at similar and low (≤ 10%) incidences in both treatment groups. Patients who experienced an increase from baseline in QTcF > 60 ms or QTcF value > 500 ms were similarly few and more frequent with moxifloxacin than lefamulin.

Among patients with elevated baseline liver enzymes and those aged ≥ 65 years, TEAEs in the hepatobiliary disorders SOC were similarly infrequent in both treatment groups (Additional file 2: Table 5). Within these patient subgroups, postbaseline elevated liver enzymes occurred in few patients, were similar with lefamulin and moxifloxacin, and resolved upon treatment discontinuation.

## Discussion

This pooled analysis confirmed lefamulin clinical efficacy and its noninferiority to moxifloxacin for treatment of adults with CABP. Outcome rates were high with lefamulin and moxifloxacin, with observed ECR rates (i.e., at 96 ± 24 h) of ~ 90% and IACR success rates at EOT, TOC, and LFU of 83.2%‒93.4%. Patients achieved ECR responder criteria early during treatment and maintained clinical response through LFU. Consistent with published literature [25–27], complete resolution of all CABP clinical signs and symptoms lagged noticeably behind ECR and cessation of antimicrobial therapy, which may be attributed to the inflammatory nature of CABP and exacerbation of preexisting conditions (e.g., asthma/COPD) or smoking-associated disability [26]. Despite this lag, however, hospitalized patients in the LEAP studies achieved clinical improvement and stability by treatment day 3 and were discharge-ready (ie, improved in ≥ 2 cardinal CABP symptoms) by day 4 [28].

Lefamulin and moxifloxacin demonstrated high and similar ECR and IACR success rates across almost all patient subgroups, with a few exceptions that may be due to small sample sizes (e.g., patients with bacteremia) or unadjusted confounders (e.g., aged < 65 years, meeting American Thoracic Society minor criteria) [29]. Complex cases of patients at risk for poor efficacy or safety outcomes due to age or comorbidity were well represented, and subpopulation analyses demonstrated high lefamulin efficacy similar to that with moxifloxacin. Similar results were observed among patients with more severe disease (i.e., PORT risk class III‒V), although clinical response rates were numerically higher in patients with PORT risk class III versus IV/V.

Lefamulin was generally well tolerated. Compared with moxifloxacin, lefamulin was associated with more frequent GI-related TEAEs; however, these were all nonserious, manageable, and rarely led to discontinuation. Patients aged ≥ 65 years or with hypertension, arrhythmia, or elevated liver enzymes are generally at greater risk of adverse safety outcomes, but these data demonstrate a safety profile for lefamulin in these patient subgroups comparable to that of the overall population. When stratified by PORT risk class, lefamulin and moxifloxacin had similar safety profiles, with higher TEAE and serious TEAE rates in patients with PORT risk class IV/V versus III, consistent with the older and more comorbid demographic of PORT risk class IV/V patients. Furthermore, the favorable safety profile observed in PORT risk class III patients suggests that they may be candidates for outpatient treatment, consistent with current consensus guidelines [30].

Extensive nonclinical testing of lefamulin suggested a potential for QT prolongation, and further assessment in phase 1 studies of healthy volunteers demonstrated dose/exposure-related QT interval effects [19]. Consistent with these findings, mild QT prolongation was seen with clinically relevant lefamulin doses in this analysis, but observed effects were consistently numerically smaller than with moxifloxacin, although the implications of QT prolongation with lefamulin should continue to be examined in clinical practice. Lefamulin is not recommended for patients taking other drugs with known QT effects [19], and experts on long QT syndrome recommend performance of an electrocardiogram before and after starting therapy with any drug(s) that has a probable or possible association with QT prolongation [31].

Potential liver injury is a concern with multiple antibiotic classes, and most (eg, cephalosporins, fluoroquinolones, macrolides, tetracyclines) list hepatic enzyme elevation and other hepatic AEs in their package inserts [32–34]. Consistent with extensive nonclinical evaluations suggesting lefamulin is unlikely to result in substantial hepatotoxicity [35], these data show a favorable lefamulin safety profile, with low frequencies of transient hepatic enzyme elevations and TEAEs. Because the liver is a major elimination route for lefamulin (primarily via CYP3A4) [35], dosage adjustment is required for IV lefamulin in patients with severe hepatic impairment; oral lefamulin has not been studied in subjects with hepatic impairment and, based on available data, is not recommended in patients with moderate or severe hepatic impairment [19].

Study findings may be limited in their generalizability, as enrollment criteria may have excluded some patients typically seen in clinical practice such as those at risk for major cardiac events or dysfunction, patients with significant hepatic disease, and patients with severely impaired renal function. However, comorbidities common in patients presenting with CABP such as age, DM, a history of cardiovascular diseases (e.g., hypertension, CHF, or arrhythmia) or chronic lung diseases (e.g., asthma or COPD), elevated liver enzymes, or mild-to-moderate renal dysfunction were well represented in our analysis. Each of these comorbidities would be important for clinicians to consider when determining the intensity of care that a given patient with CABP will require. Similar to most contemporary antimicrobial clinical trials [36, 37], enrollment in these studies was largely outside of the United States because of challenges associated with prestudy antibiotic treatment and hospitalization duration [38]. However, a recent FDA analysis of geographic differences across antimicrobial clinical trials from 2001 to 2017 indicated broad similarities in demographic, clinical, and microbiological characteristics, which lessens generalizability concerns for these trials [39]. Subgroup analysis findings are limited by relatively low sample size such that important but infrequent AEs may not have been detected and randomization may not have adequately balanced prognostic factors. Lastly, the data include some random variation owing to the number of subgroups examined.

## Conclusions

In this pooled analysis of 2 pivotal CABP studies, clinical response rates were high and similar with lefamulin and moxifloxacin, achieving rapid clinical response that was sustained through LFU. Lefamulin was generally well tolerated regardless of administration route, suggesting a favorable benefit-risk profile. Subgroup analyses suggest lefamulin may be a promising empiric monotherapy option for both inpatients and outpatients with CABP, including patients presenting with advanced age or various comorbidities. In light of rising antibacterial resistance with macrolides, tolerability concerns with other antibiotic classes (eg, fluoroquinolones, beta-lactams), and/or failure of other antibiotic options, lefamulin may provide a much-needed IV/oral empiric monotherapy alternative.

## Supplementary Information

**Additional file 1.** Independent Ethics Committees and Institutional Review Boards.

**Additional file 2.** Supplemental Tables.

## Data Availability

The datasets generated and analyzed during the current study were used under license and are therefore not publicly available. However, data are available from the authors upon reasonable request.

## Abbreviations

AE: Adverse event
ATS: American Thoracic Society
BMI: Body mass index
BUN: Blood urea nitrogen
CABP: Community-acquired bacterial pneumonia
CE: Clinically evaluable
CHF: Congestive heart failure
COPD: Chronic obstructive pulmonary disease
CrCl: Creatinine clearance
CURB-65: Confusion of new onset, BUN > 19 mg/dL, respiratory rate ≥ 30 breaths/min, systolic blood pressure < 90 mm Hg or diastolic blood pressure ≤ 60 mm Hg, and age ≥ 65 years
DM: Diabetes mellitus
ECR: Early clinical response
EOT: End of treatment
FDA: US Food and Drug Administration
GI: Gastrointestinal
IACR: Investigator assessment of clinical response
ITT: Intent to treat
IV: Intravenous
LEAP: Lefamulin Evaluation Against Pneumonia
LFU: Late follow-up
MedDRA: Medical Dictionary for Regulatory Activities
mITT: Modified intent to treat
MRSA: Methicillin-resistant *Staphylococcus aureus*
PORT: Pneumonia Outcomes Research Team
q12h: Every 12 h
q24h: Every 24 h
QTcF: QT interval corrected according to Fridericia
SIRS: Systemic inflammatory response syndrome
SOC: System organ class
TEAE: Treatment-emergent adverse event
TOC: Test of cure
WBC: White blood cell

## References

[1] Peyrani P, Mandell L, Torres A, Tillotson GS (2019). The burden of community-acquired bacterial pneumonia in the era of antibiotic resistance. Expert Rev Respir Med.

[2] McDermott KW, Elixhauser A, Sun R. Trends in hospital inpatient stays in the United States, 2005–2014: statistical brief #225. Healthcare Cost and Utilization Project (HCUP) Statistical Briefs. Rockville, MD: Agency for Healthcare Research and Quality; 2017.

[3] Xu J, Murphy SL, Kochanek KD, Arias E. Mortality in the United States, 2018. US Department of Health and Human Services, Centers for Disease Control and Prevention, National Center for Health Statistics. https://www.cdc.gov/nchs/data/databriefs/db355-h.pdf. Accessed 16 Jan 2021.

[4] Cavallazzi R, Furmanek S, Arnold FW (2020). The burden of community-acquired pneumonia requiring admission to an intensive care unit in the United States. Chest.

[5] Welte T, Torres A, Nathwani D (2012). Clinical and economic burden of community-acquired pneumonia among adults in Europe. Thorax.

[6] File TM, Marrie TJ (2010). Burden of community-acquired pneumonia in North American adults. Postgrad Med.

[7] Aujesky D, Fine MJ. The pneumonia severity index: a decade after the initial derivation and validation. Clin Infect Dis. 2008;47(suppl 3):S133–9. 10.1086/591394 18986279

[8] Amalakuhan B, Echevarria KL, Restrepo MI (2017). Managing community acquired pneumonia in the elderly - the next generation of pharmacotherapy on the horizon. Expert Opin Pharmacother.

[9] Ishiguro T, Takayanagi N, Yamaguchi S (2013). Etiology and factors contributing to the severity and mortality of community-acquired pneumonia. Intern Med.

[10] Luna CM, Palma I, Niederman MS (2016). The impact of age and comorbidities on the mortality of patients of different age groups admitted with community-acquired pneumonia. Ann Am Thorac Soc.

[11] Vardakas KZ, Kalimeris GD, Triarides NA, Falagas ME (2018). An update on adverse drug reactions related to β-lactam antibiotics. Expert Opin Drug Saf.

[12] Mason JW (2017). Antimicrobials and QT prolongation. J Antimicrob Chemother.

[13] Postma DF, Spitoni C, van Werkhoven CH (2019). Cardiac events after macrolides or fluoroquinolones in patients hospitalized for community-acquired pneumonia: post-hoc analysis of a cluster-randomized trial. BMC Infect Dis.

[14] US Food and Drug Administration. FDA drug safety communication: FDA reinforces safety information about serious low blood sugar levels and mental health side effects with fluoroquinolone antibiotics; requires label changes. https://www.fda.gov/downloads/Drugs/DrugSafety/UCM612834.pdf. Accessed 16 Jan 2021.

[15] US Food and Drug Administration. FDA drug safety communication: FDA warns about increased risk of ruptures or tears in the aorta blood vessel with fluoroquinolone antibiotics in certain patients. https://www.fda.gov/drugs/drug-safety-and-availability/fda-warns-about-increased-risk-ruptures-or-tears-aorta-blood-vessel-fluoroquinolone-antibiotics. Accessed 27 Aug 2020.

[16] Jones SC, Budnitz DS, Sorbello A, Mehta H (2013). US-based emergency department visits for fluoroquinolone-associated hypersensitivity reactions. Pharmacoepidemiol Drug Saf.

[17] Eyal Z, Matzov D, Krupkin M (2016). A novel pleuromutilin antibacterial compound, its binding mode and selectivity mechanism. Sci Rep.

[18] Schlünzen F, Pyetan E, Fucini P, Yonath A, Harms JM (2004). Inhibition of peptide bond formation by pleuromutilins: the structure of the 50S ribosomal subunit from *Deinococcus radiodurans* in complex with tiamulin. Mol Microbiol.

[19] Xenleta^™^ (lefamulin). Full prescribing information, Nabriva Therapeutics US, Inc., King of Prussia, PA; 2019.

[20] File TM, Goldberg L, Das A (2019). Efficacy and safety of intravenous-to-oral lefamulin, a pleuromutilin antibiotic, for the treatment of community-acquired bacterial pneumonia: the phase III Lefamulin Evaluation Against Pneumonia (LEAP 1) trial. Clin Infect Dis.

[21] Alexander E, Goldberg L, Das AF (2019). Oral lefamulin vs moxifloxacin for early clinical response among adults with community-acquired bacterial pneumonia: the LEAP 2 randomized clinical trial. JAMA.

[22] Paukner S, Gelone SP, Arends SJR, Flamm RK, Sader HS (2019). Antibacterial activity of lefamulin against pathogens most commonly causing community-acquired bacterial pneumonia: SENTRY antimicrobial surveillance program (2015–2016). Antimicrob Agents Chemother.

[23] Waites KB, Crabb DM, Duffy LB, et al. In vitro activities of lefamulin and other antimicrobial agents against macrolide-susceptible and macrolide-resistant *Mycoplasma pneumoniae* from the United States, Europe, and China. Antimicrob Agents Chemother. 2017;61(2):e02008–e2016. 10.1128/AAC.02008-16 PMC5278710 27855075

[24] Miettinen O, Nurminen M (1985). Comparative analysis of two rates. Stat Med.

[25] Wootton DG, Dickinson L, Pertinez H (2017). A longitudinal modelling study estimates acute symptoms of community acquired pneumonia recover to baseline by 10 days. Eur Respir J.

[26] Wyrwich KW, Yu H, Sato R, Powers JH (2015). Observational longitudinal study of symptom burden and time for recovery from community-acquired pneumonia reported by older adults surveyed nationwide using the CAP Burden of Illness Questionnaire. Patient Relat Outcome Meas.

[27] Metlay JP, Atlas SJ, Borowsky LH, Singer DE (1998). Time course of symptom resolution in patients with community-acquired pneumonia. Respir Med.

[28] Lodise T, Colman S, Stein DS (2020). Post hoc assessment of time to clinical response among adults hospitalized with community-acquired bacterial pneumonia who received either lefamulin or moxifloxacin in 2 phase III randomized, double blind, double-dummy clinical trials. Open Forum Infect Dis.

[29] File TM, Gelone SP, Schranz J, Alexander E (2020). Reply to Tang and Lai. Clin Infect Dis.

[30] Metlay JP, Waterer GW, Long AC (2019). Diagnosis and treatment of adults with community-acquired pneumonia: an official clinical practice guideline of the American Thoracic Society and Infectious Diseases Society of America. Am J Respir Crit Care Med.

[31] Al-Khatib SM, LaPointe NM, Kramer JM, Califf RM (2003). What clinicians should know about the QT interval. JAMA.

[32] Avelox^®^ (moxifloxacin hydrochloride). Full prescribing information. Bayer HealthCare Pharmaceuticals Inc., Whippany, NJ; 2019.

[33] Zithromax^®^ (azithromycin dihydrate). Full prescribing information, Pfizer Inc, New York, NY; 2019.

[34] Teflaro^®^ (ceftaroline fosamil). Full prescribing information, Allergan USA, Inc., Madison, NJ; 2019.

[35] US Food and Drug Administration, Center for Drug Evaluation and Research. Xenleta NDA/BLA multi-disciplinary review and evaluation. https://www.accessdata.fda.gov/drugsatfda_docs/nda/2019/211672Orig1s000,%20211673Orig1s000MultidisciplineR.pdf. Accessed 27 Aug 2020.

[36] Kaye KS, Bhowmick T, Metallidis S (2018). Effect of meropenem-vaborbactam vs piperacillin-tazobactam on clinical cure or improvement and microbial eradication in complicated urinary tract infection: the TANGO I randomized clinical trial. JAMA.

[37] Wagenlehner FM, Umeh O, Steenbergen J, Yuan G, Darouiche RO (2015). Ceftolozane-tazobactam compared with levofloxacin in the treatment of complicated urinary-tract infections, including pyelonephritis: a randomised, double-blind, phase 3 trial (ASPECT-cUTI). Lancet.

[38] Spellberg B, Marr KA, Brass EP (2016). Regulatory pathways for new antimicrobial agents: trade-offs to keep the perfect from being the enemy of the good. Clin Pharmacol Ther.

[39] Bart SM, Farley JJ, Bala S, Amini T, Cox E. Geographic shifts in antibacterial drug clinical trial enrollment: implications for generalizability. Clin Infect Dis. 2021;72(8):1422–8. 10.1093/cid/ciaa246 32161946

[40] Mandell LA, Wunderink RG, Anzueto A (2007). Infectious Diseases Society of America/American Thoracic Society consensus guidelines on the management of community-acquired pneumonia in adults. Clin Infect Dis.

[41] Li HY, Guo Q, Song WD (2015). Modified IDSA/ATS minor criteria for severe community-acquired pneumonia best predicted mortality. Medicine (Baltimore).

