# Supplementary material for: Lefamulin efficacy and safety in a pooled phase 3 clinical trial population with community-acquired bacterial pneumonia and common clinical comorbidities
Source: BMC Pulm Med. 2021 May 8;21:154. doi: 10.1186/s12890-021-01472-z (PMC8105923; doi:10.1186/s12890-021-01472-z)
Supplement: Supplementary file 1 — Additional file 1. Independent Ethics Committees and Institutional Review Boards. [file 12890_2021_1472_MOESM1_ESM.pdf]

Additional File 1

Supplementary Appendix 1. List of Independent Ethics Committees and Institutional Review Boards: LEAP 1 (NCT02559310)

Supplementary Appendix 2. List of Independent Ethics Committees and Institutional Review Boards: LEAP 2 (NCT02813694)

Supplementary Appendix 1. List of Independent Ethics Committees and Institutional Review Boards: LEAP 1 (NCT02559310)

Site Number	Investigator Name	Independent Ethics Committee or Institutional Review Board
1001	John Pullman, MD	Copernicus Group IRB<br>1 Triangle Drive # 100<br>Research Triangle Park, NC 27709<br>USA
1002	Hari Polenakovik, MD	Western Institutional Review Board (WIRB)<br>1019 39th Ave SE, Suite 120<br>Puyallup, WA 98374<br>USA
1004	Munib S. Daudjee, MD	Copernicus Group IRB<br>1 Triangle Drive, #100<br>Research Triangle Park, NC 27709<br>USA
1005	James Miner, MD	Minneapolis Medical Research Foundation, Human Subjects Research Committee<br>701 Park Avenue<br>Minneapolis, MN 55415<br>USA
1006	Firas A. Koura, MD, MPH, FCCP	Appalachian Regional Healthcare Inc., IRB<br>100 Airport Gardens Road<br>Hazard, KY 41701<br>USA
1008	Robert Holladay, MD	Louisiana State University Health Sciences Center-Shreveport<br>1501 Kings Highway<br>Shreveport, LA 71103<br>USA
1009	Thomas M. File, Jr., MD	Summa Health Institutional Review Board<br>525 East Market Street<br>Akron, OH 44304<br>USA
1010	Robert F. Poirier, Jr., MD, FACEP	Human Research Protection Office<br>Washington University in St. Louis<br>660 South Euclid Avenue, Campus Box 8089<br>St. Louis, MO 63110<br>USA
2001	Arnold Germar, Dr.	Veterans Memorial Medical Center Institutional Review Board<br>North Avenue, Diliman Quezon<br>Quezon City 1101<br>Philippines
2002	Joven Roque V. Gonong, MD	Institutional Ethics Review Board (LCP-IERB)<br>Lung Center of the Philippines<br>Clinical Research Facility, Quezon Ave.<br>Quezon City 1104<br>Philippines

Site Number	Investigator Name	Independent Ethics Committee or Institutional Review Board
2003	Camilo Roa, Jr., Dr	University of the Philippines Research Manila Ethics Board (UPMREB)<br>2/F Paz Mendoza Building, College of Medicine University of the Philippines Manila, 547 Pedro Gil St., Ermita<br>Manila 1000<br>Philippines
2004	Ronald Allan Payumo, Dr.	St. Luke's Institutional Ethics Review Committee<br>279 E. Rodriguez Sr. Boulevard<br>Quezon 1102<br>Philippines
2005	Ronnie Samoro, Dr.	Unified Biomedical Research Ethics Review Committee<br>West Visayas State University<br>La Paz<br>Iloilo City, Iloilo 5000<br>Philippines
2103	Weerawat Manosuthi, MD	Institutional Review Board of Bamrasnaradura Infectious Diseases Institute<br>126 Tiwanon Road, Maung<br>Nonthaburi 11000<br>Thailand
3001	Carlos Enrique Bergallo	Comité Institucional de Ética de la Investigación en Salud del Adulto<br>Avenida Patria 656<br>Córdoba X5004CDT<br>Argentina
3003	Germán Ambasch	CIEIS del Niño y del Adulto<br>Bajada Pucará 2025 1st floor<br>Córdoba 5000<br>Argentina
3004	Juan Pablo Caeiro	Comite Institutional de Etica de Investigacion en Salud (CIEIS)<br>Naciones Unidas 346<br>Friuli 2786 - Bº Parque Vélez Sarsfield<br>Cordoba X5016KEH<br>Argentina
3005	Gustavo Jorge Chaparro	Comité de Ética del Instituto Médico Platense-CEDIMP<br>Calle 51 Nº315<br>La Plata, Buenos Aires<br>1900<br>Argentina
3006	Esteban Carlos Nannini	Comité de Ética del Sanatorio Británico<br>Paraguay 40<br>Rosario, Santa Fe S2000CVB<br>Argentina

Site Number	Investigator Name	Independent Ethics Committee or Institutional Review Board
3007	Angel Ramón Minguez	Comité Institucional de Ética de a Investigación en Salud del Adulto<br>Avenida Patria 656 1ºPiso<br>Córdoba X5004CDT<br>Argentina
3008[a]	Alberto Rubén Cremona, MD	Comité de Ética en Investigación Hospital Italiano de La Plata<br>Avenida 51 Nº1725 entre calles 29 y 30<br>La Plata, Buenos Aires<br>1900<br>Argentina
3101	Suzana Margareth Ajeje Lobo, MD	Comissão Nacional de Ética em Pesquisa – CONEP<br>SEPN 510 Norte, Unidade II, Ministério da Saúde<br>Bloco "A", 3o andar, Ed. Ex-INAN<br>Brasilia, DF<br>70.450-521<br>Brazil<br><br>Comitê de Ética em Pesquisa da Faculdade de Medicina de São José do Rio Preto – FAMERP<br>Av. Brigadeiro Faria Lima, 5416<br>São José do Rio Preto, SP<br>15090-000<br>Brazil
3102	Danuza Ávila de Mello, MD	Comissão Nacional de Ética em Pesquisa – CONEP<br>SEPN 510 Norte, Unidade II, Ministério da Saúde<br>Bloco "A", 3o andar, Ed. Ex-INAN<br>Brasília, DF 70.450-521<br>Brazil<br><br>Comitê de Ética em Pesquisa da Universidade de Passo Fundo<br>Campus I – Km 171 – BR285<br>Caixa Postal 611<br>Passo Fundo, RS 99001-970<br>Brazil
3103	Maria Patelli Juliani Souza Lima, MD	Comissão Nacional de Ética em Pesquisa – CONEP<br>SEPN 510 Norte, Unidade II, Ministério da Saúde<br>Bloco "A", 3o andar, Ed. Ex-INAN<br>Brasilia, DF<br>70.450-521<br>Brazil<br><br>Comitê de Ética em Pesquisa – CEP da Pontificia Universidade Católica de Campinas – PUC/Campinas<br>Rodovia Dom Pedro I, Km 136<br>Parque das Universidades<br>Campinas, SP<br>13.086-900<br>Brazil

Site Number	Investigator Name	Independent Ethics Committee or Institutional Review Board
3104	Antonio Tarcísio de Faria Freire, MD	Comissão Nacional de Ética em Pesquisa – CONEP<br>SEPN 510 Norte, Unidade II, Ministério da Saúde<br>Bloco “A”, 3o andar, Ed. Ex-INAN<br>Brasilia, DF<br>70.450-521<br>Brazil<br><br>Comitê de Ética em Pesquisa da Santa Casa de Misericórdia de Belo Horizonte<br>Rua Domingos Vieira, 590, Santa Efigênia<br>Belo Horizonte, MG<br>CEP-30150-240<br>Brazil
3201	Cristhian Juan Galvez Vasquez	Comité Institucional de Etica en Investigación de la Asociación Benéfica Prisma<br>Calle Carlos Gonzáles 251, San Miguel<br>Lima, Lima 15088<br>Peru
3202	Manuel Jesus Mayorga Espichán	Comite Institucional de Etica en Investigación de la Asociación Benéfica Prisma<br>Calle Carlos Gonzales 251, San Miguel<br>Lima, Lima 32 15088<br>Peru
3204	Oscar Guillermo Pamo Reyna	Comite Institucional de Etica en Investigacion del Hospital Nacional Arzobispo Loayza<br>Avenida Alfonso Ugarte 848, Cercado de Lima<br>Lima, Lima 15082<br>Peru
3205	Luis Alberto Camacho Cosavalente	Comité Institucional de Etica en Investigación de la Asociación Benéfica Prisma<br>Calle Carlos Gonzáles 251, San Miguel<br>Lima, Lima 32 15088<br>Peru
4001	Sead Ahmetagic	Agency for Medicinal Products and Medical Devices<br>Veljka Mladenovica bb<br>Banja Luka<br>78000<br>Bosnia and Herzegovina
4002	Nada Koluder-Cimic	Ethics Committee of the University Clinical Center Sarajevo<br>Bolnicka 25<br>Sarajevo 71000<br>Bosnia and Herzegovina
4003	Kristina Galic	Ethics Committee of the University Clinical Hospital Mostar<br>Kralja Tvrtka bb<br>Mostar 88000<br>Bosnia and Herzegovina

Site Number	Investigator Name	Independent Ethics Committee or Institutional Review Board
4004	Lejla Calkic	Agency for Medicinal Products and Medical Devices<br>Veljka Mladenovica bb<br>Banja Luka<br>78000<br>Bosnia and Herzegovina
4101	Sotir Sotirov, Dr.	Ethics Committee for Multi-Centre Trials<br>5 Sveta Nedelya Square<br>Sofia 1000<br>Bulgaria
4102	Penka Ilieva, Dr.	Ethics Committee for Multi-Centre Trials<br>5 Sveta Nedelya Square<br>Sofia 1000<br>Bulgaria
4103	Hristo Metev, Dr.	Ethics Committee for Multi-Centre Trials<br>5 Sveta Nedelya Square<br>Sofia 1000<br>Bulgaria
4104	Margarita Taseva, Dr.	Ethics Committee for Multi-Centre Trials<br>5 Sveta Nedelya Square<br>Sofia 1000<br>Bulgaria
4105	Krasimir Donchev, Dr.	Ethics Committee for Multi-Centre Trials<br>5 Sveta Nedelya Square<br>Sofia 1000<br>Bulgaria
4106	Kalin Aleksandrov, Dr.	Ethics Committee for Multi-Centre Trials<br>5 Sveta Nedelya Square<br>Sofia 1000<br>Bulgaria
4107	Mihail Kirov, Dr.	Ethics Committee for Multi-Centre Trials<br>5 Sveta Nedelya Square<br>Sofia 1000<br>Bulgaria
4108	Grigor Lalov, Dr.	Ethics Committee for Multi-Centre Trials<br>5 Sveta Nedelya Square<br>Sofia 1000<br>Bulgaria
4109	Iveta Naydenova, Dr.	Ethics Committee for Multi-Centre Trials<br>5 Sveta Nedelya Square<br>Sofia 1000<br>Bulgaria
4110	Diana Slaveva, Dr.	Ethics Committee for Multi-Centre Trials<br>5 Sveta Nedelya Square<br>Sofia 1000<br>Bulgaria

Site Number	Investigator Name	Independent Ethics Committee or Institutional Review Board
4111	Kosta Kostov, Prof	Ethics Committee for Multi-Centre Trials<br>5 Sveta Nedelya Square<br>Sofia 1000<br>Bulgaria
4112	Galina Petrova, Dr.	Ethics Committee for Multi-Centre Trials<br>5 Sveta Nedelya Square<br>Sofia 1000<br>Bulgaria
4201	Tamaz Maglakelidze	LEC of LTD Diagnostic Services<br>36, Lubliana street<br>Tbilisi 0159<br>Georgia
4202	Rusudan Tsulaia	LTD ,,Medulla" – Chemotherapy and Immunotherapy clinic<br>6 Politkovskaia street<br>Tbilisi 0186<br>Georgia
4203	Manana Makhviladze	LTD,, Acad. Vakhtang Bochorishvili Center of Sepsis and Infection Pathology"<br>16 Aleksandre Kazbegi avenue<br>Tbilisi 0160<br>Georgia
4204	Revaz Tabukashvili	NCLE ,,Internal Medicine Clinic of Georgian Patriarchate (for indigents) and its Development Fund"<br>9 Tsinandali street<br>Tbilisi 0144<br>Georgia
4205	Gulnara Chapidze	Local Ethics Committee of LTD Emergency Cardiology Center by Academician G. Chapidze<br>4 Lubliana street<br>Tbilisi 0159<br>Georgia
4206	Kakha Vacharadze	Local Ethics Committee of LTD National Center for Tuberculosis and Lung diseases"<br>50 Maruashvili street<br>Tbilisi 0101<br>Georgia
4302	Zsolt Király, Dr.	Egészségügyi Tudományos Tanács Klinikai Farmakológiai Etikai Bizottság<br>Arany János utca 6-8<br>Budapest 1051<br>Hungary<br><br>Országos Gyógyszerészeti és Élelmezés-egészségügyi Intézet (OGYÉI)<br>Zrinyi utca 3.<br>Budapest 1051<br>Hungary

Site Number	Investigator Name	Independent Ethics Committee or Institutional Review Board
4303	Zsuzsanna Márk, Dr.	Egészségügyi Tudományos Tanács Klinikai Farmakológiai Etikai Bizottság<br>Arany János u. 6-8.<br>Budapest 1051<br>Hungary<br><br>Országos Gyógyszerészeti és Élelmezés-egészségügyi Intézet (OGYÉI)<br>Zrinyi utca 3.<br>Budapest 1051<br>Hungary
4304	István Várkonyi, Dr.	Egészségügyi Tudományos Tanács Klinikai Farmakológiai Etikai Bizottság<br>Arany János u. 6-8.<br>Budapest 1051<br>Hungary<br><br>Országos Gyógyszerészeti és Élelmezés-egészségügyi Intézet (OGYÉI)<br>Zrinyi utca 3.<br>Budapest 1051<br>Hungary
4305	Éva Francovszky, Dr.	Egészségügyi Tudományos Tanács Klinikai Farmakológiai Etikai Bizottság<br>Arany János utca 6-8<br>Budapest 1051<br>Hungary<br><br>Országos Gyógyszerészeti és Élelmezés-egészségügyi Intézet (OGYÉI)<br>Zrinyi utca 3.<br>Budapest 1051<br>Hungary
4306	Erika Unger, Dr.	Egészségügyi Tudományos Tanács Klinikai Farmakológiai Etikai Bizottság<br>Arany János u. 6-8.<br>Budapest 1051<br>Hungary<br><br>Országos Gyógyszerészeti és Élelmezés-egészségügyi Intézet (OGYÉI)<br>Zrinyi utca 3.<br>Budapest 1051<br>Hungary

Site Number	Investigator Name	Independent Ethics Committee or Institutional Review Board
4307	Lajos Molnár, Dr.	Egészségügyi Tudományos Tanács Klinikai Farmakológiai Etikai Bizottság<br>Arany János u. 6-8.<br>Budapest 1051<br>Hungary<br><br>Országos Gyógyszerészeti és Élelmezés-egészségügyi Intézet (OGYÉI)<br>Zrinyi utca 3.<br>Budapest 1051<br>Hungary
4308	Eszter Csánky, Dr.	Egészségügyi Tudományos Tanács Klinikai Farmakológiai Etikai Bizottság<br>Arany János utca 6-8<br>Budapest 1051<br>Hungary<br><br>Országos Gyógyszerészeti és Élelmezés-egészségügyi Intézet (OGYÉI)<br>Zrinyi utca 3.<br>Budapest 1051<br>Hungary
4401	Sandra Audere, Dr.	The Ethics Committee for Clinical Trials of Medicinal Products<br>Aizkraukles street 21-113<br>Riga LV-1006<br>Latvia
4402	Inga Stukena	The Ethics Committee for Clinical Trials of Medicinal Products<br>Aizkraukles street 21-113<br>Riga LV-1006<br>Latvia
4403	Olga Bogomolova, Dr.	The Ethics Committee for Clinical Trials of Medicinal Products<br>Aizkraukles street 21-113<br>Riga LV-1006<br>Latvia
4602	Willem R. Pieters	METC Maxima Medisch Centrum<br>De Run 4600<br>Veldhoven 5504 DB<br>Netherlands
4603	Hendrik Timmer<br><br>Henk Sinninghe Damste (Former PI)	METC Maxima Medisch Centrum<br>De Run 4600<br>Veldhoven<br>5504 DB<br>Netherlands

Site Number	Investigator Name	Independent Ethics Committee or Institutional Review Board
4701	Adam Antczak, MD, PhD, Prof.	Komisja Bioetyczna przy Okregowej Izbie Lekarskiej w Lodzi<br>Czerwona 3<br>Lodz, Lodzkie<br>93-005<br>Poland
4702	Malgorzata Wrobel-Rajzer, MD, PhD	Beskidzka Izba Lekarska<br>Krasinskiego 28<br>Bieslko-Biala, Slaskie 43-300<br>Poland
4703	Dariusz Jastrzebski, MD	Komisja Bioetyczna przy Okregowej Izbie Lekarskiej w Lodzi<br>Czerwona 3<br>Lodz, Lodzkie 93-005<br>Poland
4704	Zbigniew Gaciong, MD, PhD, Prof.	Komisja Bioetyczna przy Okregowej Izbie Lekarskiej w Lodzi<br>Czerwona 3<br>Lodz, Lodzkie<br>93-005<br>Poland
4801	Victoria Arama	National Bioethics Committee for Medicine and Medical Devices<br>19-21 Stefan cel Mare Road, 2nd district<br>Bucharest 020125<br>Romania
4802	Oana Cristina Arghir	National Bioethics Committee for Medicine and Medical Devices<br>19-21 Stefan cel Mare Road, 2nd district<br>Bucharest 020125<br>Romania
4803	Gheorghe Iulian Diaconescu	National Bioethics Committee for Medicine and Medical Devices<br>19-21 Stefan cel Mare Road, 2nd district<br>Bucharest 020125<br>Romania
4805	Mihaela Flavia Grosan	National Bioethics Committee for Medicine and Medical Devices<br>19-21 Stefan cel Mare Road, 2nd district<br>Bucharest 020125<br>Romania
4806	Anca Streinu-Cercel	National Bioethics Committee for Medicine and Medical Devices<br>19-21 Stefan cel Mare Road, 2nd district<br>Bucharest 020125<br>Romania
4807	Dorin Vancea	National Bioethics Committee for Medicine and Medical Devices<br>19-21 Stefan cel Mare Road, 2nd district<br>Bucharest 020125<br>Romania

Site Number	Investigator Name	Independent Ethics Committee or Institutional Review Board
4808	Mimi Floarea Nitu	National Bioethics Committee for Medicine and Medical Devices<br>19-21 Stefan cel Mare Road, 2nd district<br>Bucharest 020125<br>Romania
4809	Stefan Mihaicuta	National Bioethics Committee for Medicine and Medical Devices<br>19-21 Stefan cel Mare Road, 2nd district<br>Bucharest 020125<br>Romania
4810	Daniela Mocanescu<br><br>Simona Stefania Busca<br>(Former PI)	National Bioethics Committee for Medicine and Medical Devices<br>19-21 Stefan cel Mare Road, 2nd district<br>Bucharest 020125<br>Romania
4811	Mihaela Sorina Lupse	National Bioethics Committee for Medicine and Medical Devices<br>19-21 Stefan cel Mare Road, 2nd district<br>Bucharest 020125<br>Romania
4901	Alina Agafina	The Russian Federation Ministry of Healthcare, Department of State Regulation of Circulation of Medicines, Ethics Council<br>Rakhmanovsky Pereulok 3<br>Moscow 127994<br>Russia<br><br>Ethics Expert Council at St. Petersburg State Budget Healthcare Institution “City Hospital No. 40 of Kurortniy Administrative District<br>ulitsa Borisova, 9<br>Sestroretsk<br>St. Petersburg 197706<br>Russia
4902	Svetlana Goncharova, MD	The Russian Federation Ministry of Healthcare, Department of State Regulation of Circulation of Medicines, Ethics Council<br>Rakhmanovsky Pereulok 3<br>Moscow 127994<br>Russia<br><br>Ethics Committee at SBHI of Novosibirsk Region “City Clinical Hospital of Emergency Medicine No. 2”<br>ulitsa Turgeneva, 155<br>Novosibirsk 630008<br>Russia

Site Number	Investigator Name	Independent Ethics Committee or Institutional Review Board
4903	Alexander Gordienko	The Russian Federation Ministry of Healthcare, Department of State Regulation of Circulation of Medicines, Ethics Council<br>Rakhmanovsky Pereulok 3<br>Moscow 127994<br>Russia<br><br>Independent Ethics Committee at FSBMEI of HPE “Military Medical Academy n.a. S.M. Kirov” MD of RF<br>ulitsa Academika Lebedeva, 6<br>Saint-Petersburg 194044<br>Russia
4904	Galina Ignatova, MD, PhD, Dr.Med.Sci, Professor	The Russian Federation Ministry of Healthcare, Department of State Regulation of Circulation of Medicines, Ethics Council<br>Rakhmanovsky Pereulok 3<br>Moscow 127994<br>Russia<br><br>Ethics Committee at State Budgetary Health Institution “Regional Clinical Hospital #4”<br>ulitsa Ostrovskogo, 81<br>Chelyabinsk 454106<br>Russia
4905	Oleg Khrustalev, MD, PhD, Dr. Med. Sci	The Russian Federation Ministry of Healthcare, Department of State Regulation of Circulation of Medicines, Ethics Council<br>Rakhmanovsky Pereulok 3<br>Moscow 127994<br>Russia<br><br>Ethics Committee at State Budget Institution of Healthcare of Yaroslavl Region << Regional Clinical Hospital>><br>Ulitsa Yakovlevskaya, 7<br>Yaroslavl 150062<br>Russia
4906	Roman Kozlov, MD, PhD, Dr. Med. Sci, Professor	The Russian Federation Ministry of Healthcare, Department of State Regulation of Circulation of Medicines, Ethics Council<br>Rakhmanovsky Pereulok 3<br>Moscow 127994<br>Russia<br><br>Ethics Committee at SBEI HPE “Smolensk State Medical University” of the MoH of RF<br>ulitsa Krupskoy, 28 (legal)<br>pr. Gagarina, 27 (actual)<br>Smolensk 214019/2014018<br>Russia

Site Number	Investigator Name	Independent Ethics Committee or Institutional Review Board
5001	Zorica Lazic, Dr.	LEC of Clinical Centre Kragujevac<br>Zmaj Jovina 30<br>Kragujevac 34000<br>Serbia
5002	Branislava Milenkovic, Dr.	LEC of Clinical Centre Serbia<br>Pasterova 2<br>Belgrade 11000<br>Serbia
5003	Tatjana Pejcic, Dr.	LEC of Clinical Centre Nis<br>Bulevar dr Zorana Djindjica 48<br>Nis 18000<br>Serbia
5004	Djordje Povazan, Dr.	LEC of Institute for Pulmonary Diseases of Vojvodina<br>Put dr Goldmana 4<br>Sremska Kamenica 21204<br>Serbia
5101	Mohammed Siddique Tayob, Dr.	Pharma-Ethics Independent Research Ethics Committee<br>123 Amcor Road<br>Lyttelton Manor<br>Gauteng 0157<br>South Africa
5102	Jaco Cornelius Juhl Jurgens, Dr.	Pharma-Ethics Independent Research Ethics Committee<br>123 Amcor Road<br>Lyttelton Manor<br>Centurion, Pretoria, Gauteng<br>0157<br>South Africa
5103	Ismail Haroon Mitha, Dr.	Pharma-Ethics Independent Research Ethics Committee<br>123 Amcor Road<br>Lyttelton Manor<br>Gauteng 0157<br>South Africa
5104	Heidi Siebert, Dr.	Pharma-Ethics Independent Research Ethics Committee<br>123 Amcor Road<br>Lyttelton Manor<br>Centurion, Pretoria, Gauteng<br>0157<br>South Africa
5105	Leon Frederik Fouche, Dr.	Pharma-Ethics Independent Research Ethics Committee<br>123 Amcor Road<br>Lyttelton Manor<br>Centurion, Pretoria, Gauteng<br>0157<br>South Africa

Site Number	Investigator Name	Independent Ethics Committee or Institutional Review Board
5201	Viktor Blazhko	Commission on Ethics Questions of Municipal Institution of Health Care Kharkiv City Clinical Hospital # 13<br>137 Gagarina avenue<br>Kharkiv 61124<br>Ukraine
5202	Viktor Diachenko	Commission on Ethics Questions of Nation Military and Medical Clinical Centre “Main Military Clinical Hospital”<br>18 Hospitalna str.<br>Kyiv 01133<br>Ukraine
5203	Svitlana Kovalenko	Commission on Ethics Questions of MI “Chernivtsi Regional Clinical Hospital”<br>137 Holovna str.<br>Chernivtsi 58005<br>Ukraine
5204	Mykola Ostrovskyy	Local Commission on Bioethics Questions of Regional Centre of Phthisiology and Pulmonology<br>17 I. Franka str.<br>Ivano-Frankivsk 76018<br>Ukraine
5205	Lesya Kuryk	Commission on Ethics Questions of SI “National Institute of Phthisiology & Pulmonology n.a. F. G.Yanovskyi of NAMS of Ukraine”<br>10 Amosova str.<br>Kyiv<br>03680<br>Ukraine
5206	Ivan Vishnivetsky	Commission on Ethics Questions of Municipal Institution Central City Hospital #1 City of Zhytomyr<br>70 Velyka Berdychivska street<br>Zhytomyr 10002<br>Ukraine
5207	Liudmyla Iashyna	Commission on Ethics Questions of SI “National Institute of Phthisiology and Pulmonology n.a. F.G. Yanovskyi under NAMS of Ukraine”<br>10 Amosova str.<br>Kyiv 03680<br>Ukraine
5208	Liudmyla Prystupa	Commission on Ethics Questions of Municipal Institution of Sumy Regional Council Sumy Regional Clinical Hospital<br>48 Troitska Str.<br>Sumy 40022<br>Ukraine

Site Number	Investigator Name	Independent Ethics Committee or Institutional Review Board
5209	Natalia Golovchenko	Commission on Ethics Questions of Municipal Institution “Kherson City Clinical Hospital n.a. A. and O. Tropinykh”<br>2 Komarova Street<br>Kherson 73000<br>Ukraine
5210	Anton Sadomov	Commission on Ethics Questions of Municipal Institution “City Hospital #7”<br>9 Pryvokzalna str.<br>Zaporizhzhia 69118<br>Ukraine
5211	Oleksandr Smolianyi	Commission on Ethics Questions of Municipal Institution Odesa Regional Clinical Hospital<br>26 Zabolotnoho str.<br>Odesa 65025<br>Ukraine
5212	Roman Stets	Commission on Ethics Questions of Municipal Institute 6th City Clinical Hospital<br>34 Stalevariv Street<br>Zaporizhzhia 69035<br>Ukraine

[a]Site 3008 withdrew after the site initiation visit and before site activation.

Supplementary Appendix 2. List of Independent Ethics Committees and Institutional Review Boards: LEAP 2 (NCT02813694)

Site Number	Investigator Name	Independent Ethics Committee or Institutional Review Board
1051	Ikeadi M. Ndukwu, MD	Copernicus Group Independent Review Board<br>One Triangle Drive, Suite 100<br>Durham, NC 27713<br>USA
1052	Godson Ifeanyi Oguchi, MD	Copernicus Group Independent<br>One Triangle Drive, Suite 100<br>Durham, NC 27713<br>USA
1053	Gregory John Moran, MD	Olive View -UCLA Education & Research Institute<br>14445 Olive View Drive<br>Sylmar, CA 91342<br>USA
1054	John Pullman, MD	Copernicus Group Independent<br>One Triangle Drive, Suite 100<br>Durham, NC 27713<br>USA
1055	James Harvey Paxton, MD	Western Institutional Review Board<br>1019 39th Avenue SE<br>Suite 120<br>Puyallup, WA 98374<br>USA<br><br>Copernicus Group Independent Review Board (former)<br>One Triangle Drive, Suite 100<br>Durham, NC 27713<br>USA
1056	Richard L. Beasley, MD	Copernicus Group Independent<br>One Triangle Drive, Suite 100<br>Durham, NC 27713<br>USA
1057	Otis Barnum, DO	Copernicus Group Independent<br>One Triangle Drive, Suite 100<br>Durham, NC 27713<br>USA
1058	Robert F. Poirier Jr., MD, FACEP	Human Research Protection Office<br>Washington University in St. Louis<br>660 South Euclid Avenue<br>Campus Box 8089<br>St. Louis, MO 63110<br>USA

Site Number	Investigator Name	Independent Ethics Committee or Institutional Review Board
1059	Sara Sutherland, MD	University of Virginia Institutional Review Board for Health Sciences Research (UVA IRB-HSR)<br>One Morton Drive<br>Suite 400 Box 5<br>Charlottesville, VA 22903<br>USA
1060	Munib Shabbir Daudjee, MD	Copernicus Group Independent Review Board<br>One Triangle Drive, Suite 100<br>Durham, NC 27713<br>USA
1062	Robert Leigh Sherwin, MD	Western Institutional Review Board<br>1019 39th Avenue SE<br>Suite 120<br>Puyallup, WA 98374<br>USA<br><br>Copernicus Group Independent Review Board (former)<br>One Triangle Drive, Suite 100<br>Durham, NC 27713<br>USA
1063	Cynthia A. Mayer, DO	Chesapeake IRB<br>6940 Columbia Gateway Drive<br>Suite 110<br>Columbia, MD 21046<br>USA
1064	Jay Crossette Chanmugam, DO	Copernicus Group Independent Review Board<br>One Triangle Drive, Suite 100<br>Durham, NC 27713<br>USA
1065	Steven Stoltz, MD	Community Medical Centers Institutional Review Board<br>155 North Fresno Street, Suite 290<br>Fresno, CA 93701<br>USA
1066	Rajasekaran Annamalai, MD	Copernicus Group Independent Review Board<br>One Triangle Drive, Suite 100<br>Durham, NC 27713<br>USA
1067	Ravi K. Kamepalli, MD, FIDSA, CWS	St. Rita's Health Partners (SRHP)<br>730 West Market Street<br>Lima, OH 45801<br>USA
1068	Matthew David Sims, MD	Western Institutional Review Board<br>1019 39th Avenue SE<br>Suite 120<br>Puyallup, WA 98374<br>USA

Site Number	Investigator Name	Independent Ethics Committee or Institutional Review Board
1069	Arunkumar J. Shah, MD	Copernicus Group Independent Review Board<br>One Triangle Drive, Suite 100<br>Durham, NC 27713<br>USA
1070	Christian E. Sandrock, MD	UC Davis IRB<br>2921 Stockton Boulevard<br>Sacramento, CA 95817<br>USA
1071	Erik Geiger, MD	Copernicus Group Independent Review Board<br>One Triangle Drive, Suite 100<br>Durham, NC 27713<br>USA
1072	Augusto E. Focil, MD	Copernicus Group Independent Review Board<br>One Triangle Drive, Suite 100<br>Durham, NC 27713<br>USA
1073	Tushar Patel, MD	Copernicus Group Independent Review Board<br>One Triangle Drive, Suite 100<br>Durham, NC 27713<br>USA
1074	Paul Riska, MD	Biomedical Research Alliance of New York IRB<br>1981 Marcus Avenue, Suite 210<br>Lake Success, NY 11042<br>USA
1076	Oscar L. Hernandez, MD	Copernicus Group Independent Review Board<br>One Triangle Drive, Suite 100<br>Durham, NC 27713<br>USA
1077	Clyde O. Southwell, MD	Copernicus Group Independent Review Board<br>One Triangle Drive, Suite 100<br>Durham, NC 27713<br>USA
1078	Cherlin Johnson, MD	Copernicus Group Independent Review Board<br>One Triangle Drive, Suite 100<br>Durham, NC 27713<br>USA
1079	Cherlin Johnson, MD	Copernicus Group Independent Review Board<br>One Triangle Drive, Suite 100<br>Durham, NC 27713<br>USA
1080	Cherlin Johnson, MD	Copernicus Group Independent Review Board<br>One Triangle Drive, Suite 100<br>Durham, NC 27713<br>USA

Site Number	Investigator Name	Independent Ethics Committee or Institutional Review Board
1151	Uriel Chavarría Martínez, MD	Comité de Ética en Investigación del Hospital Universitario "Dr. José Eleuterio González"<br>Av. Francisco I. Madero y Gonzalitos Pte. S/N<br>Col. Mitras Centro<br>Monterrey, Nuevo León 64460<br>México
1152	Rodrigo Suárez Otero, MD	Comité de Ética en Investigación de Accelerium S. de R.L. de C.V.<br>Modesto Arreola número 917 Oriente<br>Esq. con Manuel Doblado<br>Col. Centro<br>Monterrey, Nuevo León 64000<br>México
1153	Francisco Marquez Diaz, MD	Comité de Ética en Investigación de Promotora Médica Aquascalientes, S.A. de C.V.<br>Ecuador Número 200<br>Col. Las Américas<br>Aguascalientes, Aguascalientes 20230<br>México
1154	Eduardo Rodríguez Noriega, MD	Comité de Ética en Investigación del Hospital Civil Fray Antonio Alcalde<br>Hospital No. 278<br>Col. El Retiro<br>Guadalajara, Jalisco 44280<br>México
2051	Arnold Germar, MD	Veterans Memorial Medical Center Institutional Review Board<br>North Avenue, Diliman<br>Quezon, National Capital Region 1100<br>Philippines
2052	Marie Grace Dawn Isidro, MD	West Visayas State University Unified Biomedical Research Ethics Review Committee<br>La Paz<br>Iloilo, Region VI 5000<br>Philippines
2053	Lalaine Llamido-Mortera, MD	Manila Central University Filemon D. Tanchoco<br>Sr. Medical Foundation Institutional Review Board (MCU-FDT IRB)<br>Samson Road<br>Caloocan City, National Capital Region 1400<br>Philippines
2054	Joel Santiaguel, MD	Hospital Ethics Committee<br>Quirino Memorial Medical Center<br>JP Rizal corner P. Tuazon Street, Project 4<br>Quezon, National Capital Region 1109<br>Philippines

Site Number	Investigator Name	Independent Ethics Committee or Institutional Review Board
2055	Albert Rafanan, MD	Chong Hua Hospital Institutional Review Board<br>Don Mariano Cui Street<br>Corner J. Llorente Street<br>Cebu, Region VII 6000<br>Philippines
2056	Joven Roque V. Gonong, MD	Lung Center of the Philippines Institutional Ethics Review Board<br>4th floor, Room 4103, Lung Center of the Philippines<br>Quezon Avenue<br>Quezon City, National Capital Region 1100<br>Philippines
2251	Sang Haak Lee, MD, PhD	Institutional Review Board of The Catholic University of Korea, St. Paul's Hospital<br>180 Wangsan-ro, Dongdaemun-gu<br>Seoul 02559<br>Korea
2252	Jung Hyun Chang, MD, PhD	Institutional Review Board of Ewha Womans University Mokdong Hospital<br>1071, Anyangcheon-ro, Yangcheon-gu<br>Seoul 07985<br>Korea
2253	Kwan Ho Lee, MD, PhD	Institutional Review Board of Yeungnam University Hospital<br>170 Hyeonchung-ro, Nam-gu<br>Daegu 42415<br>Korea
2254	Jin Woo Kim, MD	Institutional Review Board of The Catholic University of Korea, Uijeongbu St. Mary's Hospital<br>271 Cheonbo-ro<br>Uijeongbu-si, Gyeonggi-do 11765<br>Korea
2255	Kwang Ha Yoo, MD, PhD	Institutional Review Board of Konkuk University Medical Center<br>120-1 Neungdong-ro, Gwangjin-gu<br>Seoul 05030<br>Korea
2256	Joon Young Song, MD, PhD	Institutional Review Board of Korea University Guro Hospital<br>148, Gurodong-ro, Guro-gu<br>Seoul 08308<br>Korea
2257	Yonghyun Kim, MD	Institutional Review Board of The Catholic University of Korea, Bucheon St. Mary's Hospital<br>327, Sosa-ro, Wonmi-gu<br>Bucheon-si, Gyeonggi-do 14647<br>Korea
2351	Yen-Hsu Chen, MD, PhD	Kaohsiung Medical University Chung-Ho Memorial Hospital Institutional Review Board<br>No. 100, Tzyou 1st Road<br>Kaohsiung 807<br>Taiwan

Site Number	Investigator Name	Independent Ethics Committee or Institutional Review Board
2352	Yu-Feng Wei, MD	Institutional Review Board of the E-DA Hospital<br>No.6, Yida Road, Jiaosu Village, Yanchao District<br>Kaohsiung 82445<br>Taiwan
2353	Jen-Hsien Wang, MD	China Medical University & Hospital Research Ethics Committee<br>No.2, Yude Road<br>Taichung 40447<br>Taiwan
2354	Jann-Tay Wang, MD, PhD	National Taiwan University Hospital Research Ethics Committee<br>7, Chung-Shan South Road<br>Taipei 100<br>Taiwan
2355	Shih-Lung Cheng, MD, MMed, PhD	Research Ethics Review Committee<br>Far Eastern Memorial Hospital<br>No.21, Section 2, Nanya South Road, Banciao District<br>New Taipei City 220<br>Taiwan
3051	Analía Mykietiuk, MD	Comité de Ética del Instituto Médico Platense (CEDIMP)<br>Boulevard 51 No. 315/335<br>La Plata, Provincia de Buenos Aires B1900AVG<br>Argentina
3052	Carlos Enrique Bergallo, MD	Comité Institucional de Ética de la Investigación en Salud del Adulto<br>Av. Patria 656<br>Cordoba, Cordoba X5004CDT<br>Argentina
3053	Alberto Rubén Cremona, MD	Comité de Ética de la Investigación – Hospital Italiano La Plata<br>Av. 51 N° 1725<br>La Plata, Provincia de Buenos Aires B1900AXI<br>Argentina
3054	German Ambasch, MD	C.I.E.I.S del Niño y del Adulto - Polo Hospitalario<br>Bajada Pucara 2025, 1st floor<br>Cordoba, Cordoba X5000KFB<br>Argentina
3056	Pablo Eduardo Bonvehi, MD	Comité de Ética en Investigación de CEMIC<br>Av. Galván 4102<br>C.A.B.A., C.A.B.A C1431FWO<br>Argentina
3057	Angel Ramón Minguez, MD	Comité Institucional Ética de la Investigación en Salud del Adulto<br>Av. Patria 656<br>Cordoba, Cordoba X5004CDT<br>Argentina
3058	Martín Andrés Hojman, MD	Comité de Etica San Isidro – Instituto Medico de Alta Complejidad San Isidro<br>Av. del Libertador 16958<br>San Isidro, Provincia de Buenos Aires B1643CRO<br>Argentina

Site Number	Investigator Name	Independent Ethics Committee or Institutional Review Board
3059	María Cristina De Salvo, MD	Comité Independiente de Ética para Ensayos en Farmacología Clínica - CIEFC<br>Av. Cabildo 1536, 5ºB<br>C.A.B.A., C.A.B.A C1426ABP<br>Argentina
3152	Suzana Margareth Ajeje Lobo, MD, PhD	Comissão Nacional de Ética em Pesquisa - CONEP<br>SEPN 510 Norte, Bloco A 3º andar<br>Edifício Ex-IN AN, Unidade II, Ministerio da Saude<br>Brasilia, DF 70750-521<br>Brazil<br><br>Comité de Ética em Pesquisa em Seres Humanos da Faculdade de Medicina de São José do Rio Preto<br>Avenue Brigaderio Faria Lima, 5416<br>São José do Rio Preto, SP 15090-000<br>Brazil (former)
3153	Julio Cesar Stobbe, MD, PhD	Comissão Nacional de Ética em Pesquisa - CONEP<br>SEPN 510 Norte, Bloco A 3º andar<br>Edifício Ex-IN AN, Unidade II, Ministerio da Saude<br>Brasilia, DF 70750-521<br>Brazil<br><br>Comité de Ética em Pesquisa em Seres Humanos da Fundacão Universidade de Passo Fundo – VRPPG<br>BR 285 – Bairro São José<br>Campus 1, Km 171<br>Passo Fundo, RS 99052-900<br>Brazil
3154	Antonio Tarcísio de Faria Freire, MD	Comissão Nacional de Ética em Pesquisa - CONEP<br>SEPN 510 Norte, Bloco A 3º andar<br>Edifício Ex-IN AN, Unidade II, Ministerio da Saude<br>Brasilia, DF 70750-521<br>Brazil<br><br>Comitê de Ética em Pesquisa da Santa Casa de Misericórdia de Belo Horizonte<br>Rua Domingos Vieira Nº 590, Bairro Santa Efigênia<br>Belo Horizonte, MG 30150-240<br>Brazil
3251	Luis Alberto Camacho Cosavalente, MD	Comité Institucional de Ética en Investigación de la Asociación Benéfica Prisma<br>Calle Carlos Gonzales 251, Urb. Maranga<br>San Miguel<br>Lima, Lima 32<br>Peru

Site Number	Investigator Name	Independent Ethics Committee or Institutional Review Board
3252	Carlos Humberto Paz Chávez, MD	Comité Institucional de Ética en Investigación de la Asociación Benéfica Prisma<br>Calle Carlos Gonzales 251, Urb. Maranga<br>San Miguel<br>Lima, Lima 32<br>Peru
3253	Socorro Ursina Castro Bernardini, MD	Comité Institucional de Ética en Investigación de la Asociación Benéfica Prisma<br>Calle Carlos Gonzales 251, Urb. Maranga<br>San Miguel<br>Lima, Lima 32<br>Peru
3254	Lucy Melchora Gonzales Bravo, MD	Comité Institucional de Ética en Investigación de la Asociación Benéfica Prisma<br>Calle Carlos Gonzales 251, Urb. Maranga<br>San Miguel<br>Lima, Lima 32<br>Peru
3255	Rolando Alfonso Estrella Viladegut, MD	Comité Institucional de Ética en Investigación de la Asociación Benéfica Prisma<br>Calle Carlos Gonzales 251, Urb. Maranga<br>San Miguel<br>Lima, Lima 32<br>Peru
3256	Ronal Gamarra Velarde, MD	Comité Institucional de Ética en Investigación de la Asociación Benéfica Prisma<br>Calle Carlos Gonzales 251, Urb. Maranga<br>San Miguel<br>Lima, Lima 32<br>Peru
3257	Alfredo Gilberto Guerreros Benavides, MD	Comité Institucional de Ética en Investigación de la Asociación Benéfica Prisma<br>Calle Carlos Gonzales 251, Urb. Maranga<br>San Miguel<br>Lima, Lima 32<br>Peru
3258	Danilo Joel Salazar Ore, MD	Comité Institucional de Ética en la Investigación del Hospital Nacional Cayetano Heredia<br>Av. Honorio Delgado 262, Ingeniería<br>San Martín de Porres<br>Lima, Lima 31<br>Peru
3259	Juan Carlos Hinojosa Boyer, MD	Comité Institucional de Ética en Investigación de la Asociación Benéfica Prisma<br>Calle Carlos Gonzales 251, Urb. Maranga<br>San Miguel<br>Lima, Lima 32<br>Peru

Site Number	Investigator Name	Independent Ethics Committee or Institutional Review Board
3260	Zandra Judith Moncada Vilela, MD	Comité Institucional de Ética en Investigación Hospital Nacional Arzobispo Loayza<br>Avenida Alfonso Ugarte Nº 848, Cercado de Lima<br>Lima, Lima 1<br>Peru
3261	Maria Edelmira Cruz Saldarriaga, MD	Comité Institucional de Ética en Investigación de la Asociación Benéfica Prisma<br>Calle Carlos Gonzales 251, Urb. Maranga<br>San Miguel<br>Lima, Lima 32<br>Peru
3262	Remmy Ronald Flores Velarde, MD	Comité de Ética Hospital Carlos Alberto Seguín Escobedo - EsSalud<br>Calle Peral/Ayacucho S/N. Arequipa<br>Arequipa, Arequipa<br>Peru
3263	Melvin Ricalde Castro Prieto, MD	Comité Institucional de Ética en Investigación de la Asociación Benéfica Prisma<br>Calle Carlos Gonzales 251, Urb. Maranga<br>San Miguel<br>Lima, Lima 32<br>Peru
3264	Marcia Darmelly Salas Perez, MD	Comité de Ética en Investigación Biomédica del Hospital Nacional Dos de Mayo<br>Parque "Historia de la Medicina Peruana" s/n<br>Alt. Cdra.13 Av. Grau. Cercado de Lima<br>Lima, Lima<br>Peru
3265	Alberto Matsuno Fuchigami, MD	Comité Institucional de Ética en Investigación de la Asociación Benéfica Prisma<br>Calle Carlos Gonzales 251, Urb. Maranga<br>San Miguel<br>Lima, Lima 32<br>Peru
3352	Manuel Angel Muñoz Reyes, MD	Comité de Ética Científica Servicio de Salud Araucanía Sur<br>Andrés Bello #636<br>Temuco 4791301<br>Chile
3353	Juan Carlos Palma Carvajal, MD	Comité Ético Científico Servicio de Salud Metropolitano Sur<br>Avenida Santa Rosa #3453, San Miguel<br>Santiago 8900390<br>Chile
3354	Absalón Rafael Sergio Silva Orellana, MD	Comité de Ética Científico Servicio de Salud Metropolitano Oriente<br>Avenida Salvador #364, Providencia<br>Santiago 7500922<br>Chile

Site Number	Investigator Name	Independent Ethics Committee or Institutional Review Board
3355	Mario Andrés Calvo Arellano, MD	Comité Ético Científico Servicio de Salud Valdivia<br>Vicente Pérez Rosales #560<br>Edificio Prales, Oficina 307<br>Valdivia 5110537<br>Chile
3356	Andrés Isaac Rosenblut Ratinoff, MD	Comité de Ético Científico Servicio de Salud Metropolitano Sur Oriente<br>Avenida Concha y Toro #3459, Puente Alto<br>Santiago 8207257<br>Chile
3357	Sergio Luis Vargas Munita, MD	Comité de Ética de Investigación en Seres Humanos Facultad de Medicina Universidad de Chile<br>Avenida Independencia # 1027, Indepencia<br>Santiago 8380453<br>Chile<br><br>Comité de Ética de la Investigación Servicio de Salud Metropolitano Norte<br>Calle San José 1053, Independencia<br>Santiago 8380755<br>Chile
4152	Mariyana Stoyanova Markova, MD	Ethics Committee for Multicenter Trials<br>5, Sveta Nedelya Sq.<br>Sofia, Sofia 1000<br>Bulgaria
4153	Kalin Aleksandrov, MD	Ethics Committee for Multicenter Trials<br>5, Sveta Nedelya Sq.<br>Sofia, Sofia 1000<br>Bulgaria
4154	Dina Doseva, MD	Ethics Committee for Multicenter Trials<br>5, Sveta Nedelya Sq.<br>Sofia, Sofia 1000<br>Bulgaria
4156	Sonya Stoyanova-Genova, Assoc. Prof., MD	Ethics Committee for Multicenter Trials<br>5, Sveta Nedelya Sq.<br>Sofia, Sofia 1000<br>Bulgaria
4157	Ginka Kirkova, MD	Ethics Committee for Multicenter Trials<br>5, Sveta Nedelya Sq.<br>Sofia, Sofia 1000<br>Bulgaria
4158	Bistra Kostadinova, MD	Ethics Committee for Multicenter Trials<br>5, Sveta Nedelya Sq.<br>Sofia, Sofia 1000<br>Bulgaria

Site Number	Investigator Name	Independent Ethics Committee or Institutional Review Board
4159	Iveta Naydenova, MD	Ethics Committee for Multicenter Trials<br>5, Sveta Nedelya Sq.<br>Sofia, Sofia 1000<br>Bulgaria
4160	Sotir Sotirov, MD	Ethics Committee for Multicenter Trials<br>5, Sveta Nedelya Sq.<br>Sofia, Sofia 1000<br>Bulgaria
4161	Mariya Loboshka-Becheva, MD	Ethics Committe for Multicenter Trials<br>5, Sveta Nedelya Sq.<br>Sofia, Sofia 1000<br>Bulgaria
4162	Hristo Metev, MD	Ethics Committee for Multicenter Trials<br>5, Sveta Nedelya Sq.<br>Sofia, Sofia 1000<br>Bulgaria
4163	Dinko Valev, MD	Ethics Committee for Multicenter Trials<br>5, Sveta Nedelya Sq.<br>Sofia, Sofia 1000<br>Bulgaria
4164	Radka Tsenova, MD	Ethics Committee for Multicenter Trials<br>5, Sveta Nedelya Sq.<br>Sofia, Sofia 1000<br>Bulgaria
4165	Penka Ilieva, MD	Ethics Committee for Multicenter Trials<br>5, Sveta Nedelya Sq.<br>Sofia, Sofia 1000<br>Bulgaria
4252	Tamaz Maglakelidze, MD, PhD, Prof.	LEC of LTD “Acad. G. Chapidze Emergency Cardiology Center”<br>Lubliana Street N4<br>Tbilisi 0159<br>Georgia
4253	Kakha Vacharadze, MD, PhD, Prof.	LEC of JSC National Center for Tuberculosis And Lung Diseases<br>Maruashvili Street N 50<br>Tbilisi 0101<br>Georgia
4254	Luba Lagvilava, MD	LEC of LTD "Medulla” Chemotherapy And Immunotherapy Clinic<br>A. Politkovskaya Street N 6<br>Tbilisi 0186<br>Georgia
4255	Elene Sherozia, MD	LEC of “Diacor” LTD<br>Lubliana Street N5<br>Tbilisi 0159<br>Georgia

Site Number	Investigator Name	Independent Ethics Committee or Institutional Review Board
4256	David Mamuladze, MD	LEC of LTD “Unimed Adjara” Batumi Referral Hospital<br>Bagrationi Street N125<br>Batumi 6010<br>Georgia
4351	Zsuzsanna Mark, MD	Egészségügyi Tudományos Tanács Klinikai Farmakológiai Etikai Bizottsága<br>Arany János utca 6-8.<br>Budapest 1051<br>Hungary
4352	István Albert, MD	Egészségügyi Tudományos Tanács Klinikai Farmakológiai Etikai Bizottsága<br>Arany János utca 6-8.<br>Budapest 1051<br>Hungary
4353	Ákos Csomós, MD, PhD	Egészségügyi Tudományos Tanács Klinikai Farmakológiai Etikai Bizottsága<br>Arany János utca 6-8<br>Budapest 1051<br>Hungary
4354	Mária Héjja, MD	Egészségügyi Tudományos Tanács Klinikai Farmakológiai Etikai Bizottsága<br>Arany János utca 6-8.<br>Budapest 1051<br>Hungary
4451	Ivars Krastiņšm, MD	Ethics Committee for Clinical Research at Paula Stradina Clinical University Hospital Development Society<br>Pilsonu Street 13<br>Riga LV-1002<br>Latvia
4452	Anna Mironovska, MD	Ethics Committee for Clinical Research at Paula Stradina Clinical University Hospital Development Society<br>Pilsonu Street 13<br>Riga LV-1002<br>Latvia
4453	Dace Zentina, MD	Ethics Committee for Clinical Research at Paula Stradina Clinical University Hospital Development Society<br>Pilsonu Street 13<br>Riga LV-1002<br>Latvia
4551	Rodolfo Alvarez-Sala, MD	CEIC Hospital Universitari Vall d Hebron<br>Passeig de la Vall d.Hebrón s/n<br>Barcelona, Barcelona 08035<br>Spain
4552	Juan Pablo Horcajada Gallego, MD, PhD	CEIC Hospital Universitari Vall d Hebron<br>Passeig de laVall d'Hebrón s/n<br>Barcelona, Barcelona 08035<br>Spain

Site Number	Investigator Name	Independent Ethics Committee or Institutional Review Board
4553	Vicente Estrada, MD, PhD	CEIC Hospital Universitari Vall d Hebron<br>Passeig de la Vall d.Hebrón s/n<br>Barcelona, Barcelona 08035<br>Spain
4554	Mar Masia, MD, PhD	CEIC Hospital Universitari Vall d Hebron<br>Passeig de la Vall d.Hebrón s/n<br>Barcelona, Barcelona 08035<br>Spain
4555	Joaqin Burgos, MD, PhD	CEIC Hospital Universitari Vall d Hebron<br>Passeig de la Vall d.Hebrón s/n<br>Barcelona, Barcelona 08035<br>Spain
4556	Miquel Sabria, MD, PhD	CEIC Hospital Universitari Vall d Hebron<br>Passeig de la Vall d.Hebrón s/n<br>Barcelona, Barcelona 08035<br>Spain
4753	Łukasz Goliński, MD	Komisja Bioetyczna przy Okregowej Izbie Lekarskiej w Krakowie<br>ul. Krupnicza 11a<br>Kraków, Lesser Poland 31-123<br>Poland
4754	Eliza Barańska, MD	Komisja Bioetyczna przy Okregowej Izbie Lekarskiej w Krakowie<br>ul. Krupnicza 11aKraków, Poland 31-123<br>Poland
4755	Jarosław Gucwa, MD	Komisja Bioetyczna przy Okregowej Izbie Lekarskiej w Krakowie<br>ul. Krupnicza 11a<br>Kraków, Poland 31-123<br>Poland
4756	Marzena Filipowska, MD	Komisja Bioetyczna przy Okregowej Izbie Lekarskiej w Krakowie<br>ul. Krupnicza 11a<br>Kraków, Poland 31-123<br>Poland
4757	Joanna Romańczuk, MD	Komisja Bioetyczna przy Okregowej Izbie Lekarskiej w Krakowie<br>ul. Krupnicza 11a<br>Kraków, Poland 31-123<br>Poland
4851	Mihaela Malis, MD	Comisia Nationala de Bioetica a Medicamentului si a Dispozitivelor Medicale<br>Sos. Stefan Cel Mare nr 19-21<br>Sector 2<br>Bucuresti 020125<br>Romania
4853	Ruxandra Mioara Râjnoveanu, MD	Comisia Nationala de Bioetica a Medicamentului si a Dispozitivelor Medicale<br>Sos. Stefan Cel Mare nr 19-21<br>Sector 2<br>Bucuresti 020125<br>Romania

Site Number	Investigator Name	Independent Ethics Committee or Institutional Review Board
4854	Luminita Gabriela Ambert, MD	Comisia Nationala de Bioetica a Medicamentului si a Dispozitivelor Medicale<br>Sos. Stefan Cel Mare nr 19-21<br>Sector 2<br>Bucuresti 020125<br>Romania
4855	Adrian Streinu-Cercel, MD, PhD	Comisia Nationala de Bioetica a Medicamentului si a Dispozitivelor Medicale<br>Sos. Stefan Cel Mare nr 19-21<br>Sector 2<br>Bucuresti 020125<br>Romania
4856	Dorin Vancea, MD	Comisia Nationala de Bioetica a Medicamentului si a Dispozitivelor Medicale<br>Sos. Stefan Cel Mare nr 19-21<br>Sector 2<br>Bucuresti 020125<br>Romania
4857	Floarea Mimi Niţu, MD, PhD	Comisia Nationala de Bioetica a Medicamentului si a Dispozitivelor Medicale<br>Sos. Stefan Cel Mare nr 19-21<br>Sector 2<br>Bucuresti 020125<br>Romania
4858	Costel Sorin Stamate, MD	Comisia Nationala de Bioetica a Medicamentului si a Dispozitivelor Medicale<br>Sos. Stefan Cel Mare nr 19-21<br>Sector 2<br>Bucuresti 020125<br>Romania
4859	Diana Ileana Glontescu, MD	Comisia Nationala de Bioetica a Medicamentului si a Dispozitivelor Medicale<br>Sos. Stefan Cel Mare nr 19-21<br>Sector 2<br>Bucuresti 020125<br>Romania
4951	Boris Goloschekin, MD, PhD	EC at Saint-Petersburg State Budget Healthcare Institution "City Hospital #15"<br>4 Avangardnaya str.<br>St- Petersburg 198205<br>Russia
4952	Alexander Solomatin, MD	EC at Federal Budget Healthcare Institution "Medical Rehabilitation Centre of the Ministry of Economic Development of the RF"<br>43 Lomonosovsky Prospect<br>Moscow 119192<br>Russia

Site Number	Investigator Name	Independent Ethics Committee or Institutional Review Board
4953	Tatiana Martynenko, MD, PhD	Local Ethics Committee at Regional State Budgetary Healthcare Institution “City Hospital #5, Barnaul”<br>75 Zmeinogorsky trakt<br>Barnaul 656045<br>Russia
4954	Lyubov Shpagina, MD, PhD, DMSc, Prof.	EC at Municipal Budgetary Healthcare Institution of Novosibirsk “City Clinical Hospital No.2”<br>21 Polzunova street<br>Novosibirsk 630051<br>Russia
4955	Vladimir Simanenkov, MD, PhD, DMSc, Prof.	EC at Saint Petersburg State Budget Healthcare Institution City Hospital No.26<br>2 Kostyushko str.<br>Saint-Petersburg 196247<br>Russia
4957	Ivan Gordeev, MD, PhD	Ethics Committee of Russian National Research Medical University n.a. N.I.Pirogov<br>1, Ostrovityanova str.<br>Moscow 117997<br>Russia
4958	Roman Kozlov, MD, PhD, DMSc, Prof.	IEC at FSBEI HE Smolensk State Medical University of the Ministry of Healthcare of the Russian Federation<br>28 Krupskoy str.<br>Smolensk 214019<br>Russia
4959	Olga Reshetko, MD, DMSc, Prof.	EC at State Healthcare Institution Regional Clinical Hospital<br>1 Smirnovskoye Ushchelye<br>Saratov 410053<br>Russia
5051	Vojislav Radosavljević, MD	Ethics Committee of Clinical Hospital Center "Bezanijska Kosa"<br>Bezanijska Kosa bb<br>Belgrade 11080<br>Serbia
5052	Violeta Mihailovic-Vucinic, MD	Ethics Committee of Clinical Center of Serbia<br>Pasterova 2<br>Belgrade 11000<br>Serbia
5053	Djordje Povazan, MD	Ethics Committee of Institute for Pulmonary Diseases of Vojvodina<br>Put dr Goldmana 4<br>Sremska Kamenica 21204<br>Serbia
5054	Zorica Lazić, MD	Ethics Committee of Clinical Center Kragujevac<br>Zmaj Jovina 30<br>Kragujevac 34000<br>Serbia

Site Number	Investigator Name	Independent Ethics Committee or Institutional Review Board
5055	Tatjana Pejčić, MD	Ethics Committee of Clinical Center Niš<br>Bulevar Zorana Djindjica 48<br>Niš 18000<br>Serbia
5056	Slobodan Aćimović, MD	Ethics Committee of Military Medical Academy<br>Crnotravska 17<br>Belgrade 11000<br>Serbia
5057	Vesna Dopudja Pantic, MD	Ethics Committee of Clinical Hospital Center "Zvezdara"<br>Dimitrija Tucovica 161<br>Belgrade 11050<br>Serbia
5151	Johannes Jurgens Lombaard, MBChB	The South African Medical Association<br>Block F, Castle Walk Office Park<br>Nossob Street, Erasmuskloof, Ext. 3<br>Pretoria, Gauteng 0183<br>South Africa
5152	Muhammed Ameen Fulat, MBChB	The South African Medical Association<br>Block F, Castle Walk Office Park<br>Nossob Street, Erasmuskloof, Ext. 3<br>Pretoria, Gauteng 0183<br>South Africa
5153	Elizabeth Catherine Wessels, MBChB	The South African Medical Association<br>Block F, Castle Walk Office Park<br>Nossob Street, Erasmuskloof, Ext. 3<br>Pretoria, Gauteng 0183<br>South Africa
5154	Jaco Cornelius Juhl Jurgens, MBChB	The South African Medical Association<br>Block F, Castle Walk Office Park<br>Nossob Street, Erasmuskloof, Ext. 3<br>Pretoria, Gauteng 0183<br>South Africa
5155	Mohammed Siddique Tayob, MBChB	The South African Medical Association<br>Block F, Castle Walk Office Park<br>Nossob Street, Erasmuskloof, Ext. 3<br>Pretoria, Gauteng 0183<br>South Africa
5156	Johannes Breedt, MBChB	The South African Medical Association<br>Block F, Castle Walk Office Park<br>Nossob Street, Erasmuskloof, Ext. 3<br>Pretoria, Gauteng 0183<br>South Africa
5251	Oleksandr Dzyublyk, DMSc, Prof.	LEC of the State Institution "National Institute of Phthisiatry and Pulmonology named after F.G. Yanovski of the NAMS of Ukraine"<br>10, Amosova str.<br>Kyiv 03680<br>Ukraine

Site Number	Investigator Name	Independent Ethics Committee or Institutional Review Board
5252	Vasyl Melnyk, DMSc, Prof.	LEC of Kyiv City Tuberculosis Hospital No.1 with Dispensary Department<br>121/3 Kharkivske shosse<br>Kyiv 02091<br>Ukraine
5253	Roman Stets, MD, PhD	LEC of the Public Institution "6th City Clinical Hospital"<br>34 Stalevariv Str.<br>Zaporizhzhia 69035<br>Ukraine
5254	Nadiya Tryshchuk. MD, PhD	Local Ethics Committee within the Educational Scientific-Practice Medical Complex<br>4, Tekstylna Street<br>Kharkiv 61157<br>Ukraine
5255	Liliya Byelaya, MD	Ethics Committee at Public Institution "Kherson City Clinical Hospital named after Ie. Ie. Karabelesha"<br>22/1 Ushakova Avenue<br>Kherson 73000<br>Ukraine
5256	Lyubov Voyeykova, CMSc	LEC of the Public Healthcare Institution "Kharkiv City Clinical Hospital No.13"<br>137, Gagarina pr.<br>Kharkiv 61124<br>Ukraine
5257	Volodymyr Koshlia, DMSc, Prof., Honored Scientist of Ukraine	LEC of the Public Institution "Zaporizhzhia City Multidisciplinary Clinical Hospital No.9"<br>1, Shchaslyva vul<br>Zaporizhzhia 69065<br>Ukraine
5258	Vasyl Neyko, DMSc, Prof.	Ethics Committee at Ivano-Frankivsk Central City Clinical Hospital<br>114 Mazepy vul.<br>Ivano-Frankivsk 76025<br>Ukraine
5259	Igor Kaydashev, DMSc	Ethics Committee at 1st City Clinical Hospital<br>Str. Olesya Honchara 27a<br>Poltava 36038<br>Ukraine
5260	Yuriy Mostovoy, DMSc, Prof.	Ethics Committee at City Clinical Hospital No.1<br>96, Khmelnytske Shose<br>Vinnytsia 21029<br>Ukraine
5261	Mykola Ostrovskyy, DMSc, Prof.	LEC of the Regional Phthisiopulmonology Center<br>17 Franka vul.<br>Ivano-Frankivsk 76018<br>Ukraine

Site Number	Investigator Name	Independent Ethics Committee or Institutional Review Board
5263	Viktor Diachenko, CMSc	LEC of the NMCC “Main Military Clinical Hospital” of the MD of Ukraine, Pulmonology Clinic (with wards for chemotheraphy)<br>18 Hospitalna vul.<br>Kyiv 01133<br>Ukraine
5264	Hanna Stupnytska, CMSc	LEC of the Regional Public Institution “Chernivtsi Regional Clinical Hospital”<br>137 Holovna Street<br>Chernivtsi 58001<br>Ukraine
5265	Liudmyla Yashyna, DMSc, Prof.	LEC of the State Institution “National Institute of Phthisiatry and Pulmonology” named after F.G. Yanovski of the NAMS of Ukraine<br>10, Amosova str.<br>Kyiv 03680<br>Ukraine
