# Supplementary material for: Lefamulin efficacy and safety in a pooled phase 3 clinical trial population with community-acquired bacterial pneumonia and common clinical comorbidities
Source: BMC Pulm Med. 2021 May 8;21:154. doi: 10.1186/s12890-021-01472-z (PMC8105923; doi:10.1186/s12890-021-01472-z)
Supplement: Supplementary file 2 — Additional file 2. Supplemental Tables. [file 12890_2021_1472_MOESM2_ESM.pdf]

Additional File 2

Supplementary Table 1. Definitions of Medical History Terms

Supplementary Table 2. Summary of TEAEs in Medical History Patient Subgroups (Pooled Safety Population)

Supplementary Table 3. Summary of TEAEs in Baseline Liver Enzyme Elevation or Renal Impairment Subgroups (Pooled Safety Population)

Supplementary Table 4. Summary of TEAEs in Age Group and by PORT Risk Class Subgroups (Pooled Safety Population)

Supplementary Table 5. Cardiac TEAEs and Postbaseline Electrocardiogram Changes in Patients at Risk of Cardiac Safety Concerns (Pooled Safety Population)

Supplementary Table 6. Hepatobiliary TEAEs and Postbaseline Liver Enzyme Changes in Patients at Risk of Hepatic Safety Concerns (Pooled Safety Population)

Supplementary Table 1. Definitions of Medical History Terms

Medical History Term	Definition
Arrhythmia	MedDRA HLGT “cardiac arrhythmias”
Asthma/COPD	MedDRA HLT “bronchospasm and obstruction”
Baseline liver enzyme elevation	Baseline AST or ALT value >ULN
Congestive heart failure	MedDRA PTs “cardiac failure,” “cardiac failure congestive,” or “cardiac failure chronic”
Diabetes mellitus	MedDRA HLT “diabetes mellitus (incl subtypes)”
Hypertension	MedDRA HLT “vascular hypertensive disorders NEC”

ALT = alanine aminotransferase; AST = aspartate aminotransferase; COPD = chronic obstructive pulmonary disease; HLGT = high-level group term; HLT = high-level term; MedDRA = Medical Dictionary for Regulatory Activities; PT = preferred term; ULN = upper limit of normal.

Supplementary Table 2. Summary of TEAEs in Medical History Patient Subgroups (Pooled Safety Population)

	Hypertension History		Asthma/COPD History		Diabetes Mellitus History		CHF History		Arrhythmia History	
**Patients, n (%)**	**Lefamulin (n=246)**	**Moxifloxacin (n=252)**	**Lefamulin (n=118)**	**Moxifloxacin (n=113)**	**Lefamulin (n=80)**	**Moxifloxacin (n=87)**	**Lefamulin (n=55)**	**Moxifloxacin (n=75)**	**Lefamulin (n=42)**	**Moxifloxacin (n=30)**
All TEAEs	86 (35.0)	80 (31.7)	42 (35.2)	43 (38.1)	29 (36.3)	29 (33.3)	20 (36.4)	28 (37.3)	20 (47.6)	14 (46.7)
Mild*	43 (17.5)	42 (16.7)	14 (11.9)	23 (20.4)	16 (20.0)	19 (21.8)	10 (18.2)	11 (14.7)	8 (19.0)	4 (13.3)
Moderate*	30 (12.2)	27 (10.7)	20 (16.9)	15 (13.3)	8 (10.0)	6 (6.9)	8 (14.5)	10 (13.3)	9 (21.4)	5 (16.7)
Severe*	13 (5.3)	11 (4.4)	8 (6.8)	5 (4.4)	5 (6.3)	4 (4.6)	2 (3.6)	7 (9.3)	3 (7.1)	5 (16.7)
Related TEAEs†	31 (12.6)	29 (11.5)	16 (13.6)	16 (14.2)	12 (15.0)	9 (10.3)	7 (12.7)	11 (14.7)	8 (19.0)	6 (20.0)
Serious TEAEs‡	16 (6.5)	14 (5.6)	13 (11.0)	6 (5.3)	4 (5.0)	7 (8.0)	4 (7.3)	7 (9.3)	4 (9.5)	5 (16.7)
TEAEs leading to study drug discontinuation§	7 (2.8)	11 (4.4)	3 (2.5)	7 (6.2)	2 (2.5)	2 (2.3)	0	6 (8.0)	0	2 (6.7)
TEAEs leading to death (by study day 28)	3 (1.2)	4 (1.6)	2 (1.7)	3 (2.7)	2 (2.5)	2 (2.3)	0	3 (4.0)	0	0
TEAEs leading to death (over entire study duration)‖	6 (2.4)	4 (1.6)	5 (4.2)	3 (2.7)	2 (2.5)	2 (2.3)	0	3 (4.0)	0	1 (3.3)

See **Supplementary Table 1** for medical history term definitions.

CHF = congestive heart failure; COPD = chronic obstructive pulmonary disease; PORT = Pneumonia Outcomes Research Team; TEAE = treatment-emergent adverse event.

*If a patient experienced a TEAE multiple times with different levels of severity, the patient was counted only once under the highest severity.

†Related TEAEs were defined as TEAEs that were considered "definitely," "probably," or "possibly" related to study drug by the investigator. If the relationship for a TEAE was missing, it was considered "related." Patients with multiple events in each category were counted only once in that category.

‡A TEAE was classified as serious if it was life threatening, resulted in death or persistent or significant disability/incapacity, required inpatient hospitalization or prolongation of existing hospitalization, or was a congenital anomaly/birth defect or an important medical event that jeopardized the patient or required medical/surgical intervention.

§A patient could have had >1 TEAE leading to study drug discontinuation.

‖Three patients in the lefamulin group had a TEAE leading to death after study day 28: 1 patient (lefamulin group; aged 87 years; PORT risk class III; liver enzyme elevation and moderate renal impairment [creatinine clearance 30 to <60 mL/min] at baseline; history of hypertension and COPD) died on study day 32 from sepsis (first reported on study day 31); 1 patient (lefamulin group; aged 80 years; PORT risk class III; baseline moderate renal impairment; history of hypertension and COPD) died on study day 57 from endocarditis (first reported on study day 24); and 1 patient (lefamulin group; aged 70 years; PORT risk class II; baseline moderate renal impairment; history of hypertension and COPD) died on study day 271 from acute myeloid leukemia (first reported on study day 269). One patient in the moxifloxacin group (aged 26 years; PORT risk class IV) died on study day 48 from testicular seminoma (first reported on study day 21).

Supplementary Table 3. Summary of TEAEs in Baseline Liver Enzyme Elevation or Renal Impairment Subgroups (Pooled Safety Population)

	Baseline Liver Enzyme Elevation		Renal Impairment							
			None		Mild		Moderate		Severe	
Patients, n (%)	Lefamulin (n=119)	Moxifloxacin (n=144)	Lefamulin (n=310)	Moxifloxacin (n=311)	Lefamulin (n=198)	Moxifloxacin (n=192)	Lefamulin (n=125)	Moxifloxacin (n=132)	Lefamulin (n=7)	Moxifloxacin (n=6)
All TEAEs	50 (42.0)	55 (38.2)	103 (33.2)	81 (26.0)	67 (33.8)	63 (32.8)	50 (40.0)	48 (36.4)	4 (57.1)	3 (50.0)
Mild*	28 (23.5)	29 (20.1)	61 (19.7)	51 (16.4)	32 (16.2)	42 (21.9)	24 (19.2)	22 (16.7)	2 (28.6)	2 (33.3)
Moderate*	15 (12.6)	18 (12.5)	36 (11.6)	24 (7.7)	27 (13.6)	15 (7.8)	15 (12.0)	16 (12.1)	0	0
Severe*	7 (5.9)	8 (5.6)	6 (1.9)	6 (1.9)	8 (4.0)	6 (3.1)	11 (8.8)	10 (7.6)	2 (28.6)	1 (16.7)
Related TEAEs†	17 (14.3)	19 (13.2)	52 (16.8)	26 (8.4)	22 (11.1)	21 (10.9)	25 (20.0)	20 (15.2)	0	1 (16.7)
Serious TEAEs‡	9 (7.6)	12 (8.3)	8 (2.6)	8 (2.6)	13 (6.6)	10 (5.2)	14 (11.2)	12 (9.1)	1 (14.3)	1 (16.7)
TEAEs leading to study drug discontinuation§	5 (4.2)	6 (4.2)	4 (1.3)	6 (1.9)	7 (3.5)	8 (4.2)	8 (6.4)	7 (5.3)	1 (14.3)	0
TEAEs leading to death (by study day 28)	2 (1.7)	4 (2.8)	0	0	4 (2.0)	2 (1.0)	4 (3.2)	4 (3.0)	0	1 (16.7)
TEAEs leading to death (over entire study duration)‖	3 (2.5)	4 (2.8)	0	1 (0.3)	4 (2.0)	2 (1.0)	7 (5.6)	4 (3.0)	0	1 (16.7)

See **Supplementary Table 1** for medical history term definitions.

COPD = chronic obstructive pulmonary disease; PORT = Pneumonia Outcomes Research Team; TEAE = treatment-emergent adverse event.

*If a patient experienced a TEAE multiple times with different levels of severity, the patient was counted only once under the highest severity.

†Related TEAEs were defined as TEAEs that were considered "definitely," "probably," or "possibly" related to study drug by the investigator. If the relationship for a TEAE was missing, it was considered "related." Patients with multiple events in each category were counted only once in that category.

‡A TEAE was classified as serious if it was life threatening, resulted in death or persistent or significant disability/incapacity, required inpatient hospitalization or prolongation of existing hospitalization, or was a congenital anomaly/birth defect or an important medical event that jeopardized the patient or required medical/surgical intervention.

§A patient could have had >1 TEAE leading to study drug discontinuation.

‖Three patients in the lefamulin group had a TEAE leading to death after study day 28: 1 patient (lefamulin group; aged 87 years; PORT risk class III; liver enzyme elevation and moderate renal impairment [creatinine clearance 30 to <60 mL/min] at baseline; history of hypertension and COPD) died on study day 32 from sepsis (first reported on study day 31); 1 patient (lefamulin group; aged 80 years; PORT risk class III; baseline moderate renal impairment; history of hypertension and COPD) died on study day 57 from endocarditis (first reported on study day 24); and 1 patient (lefamulin group; aged 70 years; PORT risk class II; baseline moderate renal impairment; history of hypertension and COPD) died on study day 271 from acute myeloid leukemia (first reported on study day 269). One patient in the moxifloxacin group (aged 26 years; PORT risk class IV) died on study day 48 from testicular seminoma (first reported on study day 21).

Supplementary Table 4. Summary of TEAEs in Age Group and by PORT Risk Class Subgroups (Pooled Safety Population)

	Age Group								PORT Risk Class					
	18–64 years		65–74 years		75–84 years		≥85 years		I/II		III		IV/V	
**Patients, n (%)**	**Lefamulin (n=374)**	**Moxifloxacin (n=393)**	**Lefamulin (n=152)**	**Moxifloxacin (n=145)**	**Lefamulin (n=89)**	**Moxifloxacin (n=86)**	**Lefamulin (n=26)**	**Moxifloxacin (n=17)**	**Lefamulin (n=184)**	**Moxifloxacin (n=192)**	**Lefamulin (n=337)**	**Moxifloxacin (n=333)**	**Lefamulin (n=120)**	**Moxifloxacin (n=116)**
All TEAEs	143 (38.2)	115 (29.3)	34 (22.4)	46 (31.7)	36 (40.4)	30 (34.9)	11 (42.3)	4 (23.5)	72 (39.1)	46 (24.0)	97 (28.8)	98 (29.4)	55 (45.8)	51 (44.0)
Mild*	79 (21.1)	73 (18.6)	15 (9.9)	26 (17.9)	16 (18.0)	16 (18.6)	9 (34.6)	2 (11.8)	39 (21.2)	29 (15.1)	56 (16.6)	62 (18.6)	24 (20.0)	26 (22.4)
Moderate*	53 (14.2)	32 (8.1)	13 (8.6)	16 (11.0)	12 (13.5)	7 (8.1)	0	0	28 (15.2)	15 (7.8)	32 (9.5)	26 (7.8)	18 (15.0)	14 (12.1)
Severe*	11 (2.9)	10 (2.5)	6 (3.9)	4 (2.8)	8 (9.0)	7 (8.1)	2 (7.7)	2 (11.8)	5 (2.7)	2 (1.0)	9 (2.7)	10 (3.0)	13 (10.8)	11 (9.5)
Related TEAEs†	63 (16.8)	39 (9.9)	17 (11.2)	18 (12.4)	17 (19.1)	9 (10.5)	2 (7.7)	2 (11.8)	39 (21.2)	16 (8.3)	37 (11.0)	37 (11.1)	23 (19.2)	15 (12.9)
Serious TEAEs‡	15 (4.0)	16 (4.1)	8 (5.3)	5 (3.4)	11 (12.4)	9 (10.5)	2 (7.7)	1 (5.9)	9 (4.9)	4 (2.1)	12 (3.6)	14 (4.2)	15 (12.5)	13 (11.2)
TEAEs leading to study drug discontinuation§	12 (3.2)	8 (2.0)	4 (2.6)	5 (3.4)	4 (4.5)	6 (7.0)	0	2 (11.8)	3 (1.6)	5 (2.6)	8 (2.4)	8 (2.4)	9 (7.5)	8 (6.9)
TEAEs leading to death (by study day 28)	2 (0.5)	2 (0.5)	3 (2.0)	2 (1.4)	3 (3.4)	3 (3.5)	0	0	0	0	3 (0.9)	2 (0.6)	5 (4.2)	5 (4.3)
TEAEs leading to death (over entire study duration)‖	2 (0.5)	3 (0.8)	4 (2.6)	2 (1.4)	4 (4.5)	3 (3.5)	1 (3.8)	0	1 (0.5)	0	5 (1.5)	2 (0.6)	5 (4.2)	6 (5.2)

COPD = chronic obstructive pulmonary disease; PORT = Pneumonia Outcomes Research Team; TEAE = treatment-emergent adverse event.

*If a patient experienced a TEAE multiple times with different levels of severity, the patient was counted only once under the highest severity.

†Related TEAEs were defined as TEAEs that were considered “definitely,” “probably,” or “possibly” related to study drug by the investigator. If the relationship for a TEAE was missing, it was considered “related.” Patients with multiple events in each category were counted only once in that category.

‡A TEAE was classified as serious if it was life threatening, resulted in death or persistent or significant disability/incapacity, required inpatient hospitalization or prolongation of existing hospitalization, or was a congenital anomaly/birth defect or an important medical event that jeopardized the patient or required medical/surgical intervention.

§A patient could have had >1 TEAE leading to study drug discontinuation.

‖Three patients in the lefamulin group had a TEAE leading to death after study day 28: 1 patient (lefamulin group; aged 87 years; PORT risk class III; liver enzyme elevation and moderate renal impairment [creatinine clearance 30 to <60 mL/min] at baseline; history of hypertension and COPD) died on study day 32 from sepsis (first reported on study day 31); 1 patient (lefamulin group; aged 80 years; PORT risk class III; baseline moderate renal impairment; history of hypertension and COPD) died on study day 57 from endocarditis (first reported on study day 24); and 1 patient (lefamulin group; aged 70 years; PORT risk class II; baseline moderate renal impairment; history of hypertension and COPD) died on study day 271 from acute myeloid leukemia (first reported on study day 269). One patient in the moxifloxacin group (aged 26 years; PORT risk class IV) died on study day 48 from testicular seminoma (first reported on study day 21).

Supplementary Table 5. Cardiac TEAEs and Postbaseline Electrocardiogram Changes in Patients at Risk of Cardiac Safety Concerns (Pooled Safety Population)

	Patients with history of hypertension, n (%)		Patients with history of arrhythmia, n (%)		Patients aged ≥65 years, n (%)	
**Parameter**	**Lefamulin (n=246)**	**Moxifloxacin (n=252)**	**Lefamulin (n=42)**	**Moxifloxacin (n=30)**	**Lefamulin (n=267)**	**Moxifloxacin (n=248)**
TEAEs in cardiac disorders SOC	8 (3.3)*	8 (3.2)*	4 (9.5)†	3 (10.0)†	3 (1.1)‡	3 (1.2)‡
TE-AESIs in QT prolongation category§	1 (0.4)	4 (1.6)	2 (4.8)	1 (3.3)	3 (1.1)	3 (1.2)
Patients with both baseline and postbaseline values of QTcF	**(n=244)**	**(n=251)**	**(n=42)**	**(n=30)**	**(n=266)**	**(n=247)**
Increase in QTcF	215 (88.1)	223 (88.8)	36 (85.7)	22 (73.3)	234 (88.0)	218 (88.3)
Increase >30 msec in QTcF	45 (18.4)	57 (22.7)	10 (23.8)	8 (26.7)	52 (19.5)	49 (19.8)
Increase >60 msec in QTcF	4 (1.6)	8 (3.2)	1 (2.4)	3 (10.0)	4 (1.5)	7 (2.8)
Value QTcF >480 msec	10 (4.1)	9 (3.6)	2 (4.8)	3 (10.0)	11 (4.1)	14 (5.7)
Value QTcF >500 msec	1 (0.4)	2 (0.8)	0	1 (3.3)	1 (0.4)	6 (2.4)
Baseline QTcF ≤480 msec and postbaseline QTcF >480 msec	9 (3.7)	7 (2.8)	2 (4.8)	2 (6.7)	10 (3.8)	10 (4.0)
Baseline QTcF ≤500 msec and postbaseline QTcF >500 msec	1 (0.4)	1 (0.4)	0	0	1 (0.4)	4 (1.6)

See **Supplementary Table 1** for medical history term definitions.

QTcF = QT interval corrected according to Fridericia; SMQ = Standardized Medical Dictionary for Regulatory Activities query; SOC = system organ class; TEAE = treatment-emergent adverse event; TE-AESI = treatment-emergent adverse event of special interest.

*Specific preferred terms that occurred in >1 patient were myocardial infarction (lefamulin, n=2), acute myocardial infarction (moxifloxacin, n=2), and atrial fibrillation (moxifloxacin, n=3); all other cardiac TEAEs occurred in ≤1 patient per treatment group.

†Specific preferred term that occurred in >1 patient was atrial fibrillation (lefamulin, n=2); all other cardiac TEAEs occurred in ≤1 patient per treatment group.

‡All cardiac TEAEs occurred in ≤1 patient per treatment group.

§Included broad SMQ search for "Torsades des Pointes/QT Prolongation."

Supplementary Table 6. Hepatobiliary TEAEs and Postbaseline Liver Enzyme Changes in Patients at Risk of Hepatic Safety Concerns (Pooled Safety Population)

	Patients with baseline liver enzyme elevation		Patients aged ≥65 y	
**Parameter**	**Lefamulin (n=119)**	**Moxifloxacin (n=144)**	**Lefamulin (n=267)**	**Moxifloxacin (n=248)**
TEAEs in hepatobiliary SOC,* n (%)	4 (3.4)	3 (2.1)	2 (0.7)	1 (0.4)
TE-AESIs in liver safety,† n (%)	2 (1.7)	9 (6.3)	5 (1.9)	6 (2.4)
Any postbaseline value, n/N (%)				
ALT >3× ULN	2/36 (5.6)	5/34 (14.7)	11/262 (4.2)	8/242 (3.3)
ALT >5× ULN	1/36 (2.8)	1/34 (2.9)	3/262 (1.1)	4/242 (1.7)
ALT >10× ULN	0/36	0/34	1/262 (0.4)	0/242
AST >3× ULN	0/23	0/39	6/262 (2.3)	4/242 (1.7)
AST >5× ULN	0/23	0/39	2/262 (0.8)	2/242 (0.8)
AST >10× ULN	0/23	0/39	1/262 (0.4)	0/242
Total bilirubin value >2× ULN	1/102 (1.0)	1/124 (0.8)	0/262	1/242 (0.4)
ALT or AST >3× ULN and total bilirubin value >2× ULN	0/55	1/64 (1.6)	0/262	1/242 (0.4)

See **Supplementary Table 1** for medical history term definitions.

ALT = alanine aminotransferase; AST = aspartate aminotransferase; SMQ = Standardized Medical Dictionary for Regulatory Activities query; SOC = system organ class; TEAE = treatment-emergent adverse event; TE-AESI = treatment-emergent adverse event of special interest; ULN = upper limit of normal.

*All hepatobiliary TEAEs occurred in ≤1 patient per treatment group.

†TE-AESIs in the liver safety SMQ included broad searches for "liver-related investigations, signs, symptoms" and "biliary-related investigations, signs, symptoms."
